# Development of a Selective Peptide κ-Opioid Receptor Antagonist by Late-Stage Functionalization with Cysteine Staples

**DOI:** 10.1021/acs.jmedchem.3c00426

**Published:** 2023-08-26

**Authors:** Edin Muratspahić, Andrew M. White, Cosmin I. Ciotu, Nadine Hochrainer, Nataša Tomašević, Johannes Koehbach, Richard J. Lewis, Mariana Spetea, Michael J. M. Fischer, David J. Craik, Christian W. Gruber

**Affiliations:** † Center for Physiology and Pharmacology, Institute of Pharmacology, Medical University of Vienna, 1090 Vienna, Austria; ‡ Institute for Molecular Bioscience, Australian Research Council Centre of Excellence for Innovations in Peptide and Protein Science, The University of Queensland, 4072 Brisbane, Queensland, Australia; § Center for Physiology and Pharmacology, Institute of Physiology, Medical University of Vienna, 1090 Vienna, Austria; ∥ Department of Pharmaceutical Chemistry, Institute of Pharmacy and Center for Molecular Biosciences Innsbruck (CMBI), University of Innsbruck, Innrain 80-82, 6020 Innsbruck, Austria; ⊥ Institute for Molecular Bioscience, The University of Queensland, 4072 Brisbane, Queensland, Australia

## Abstract

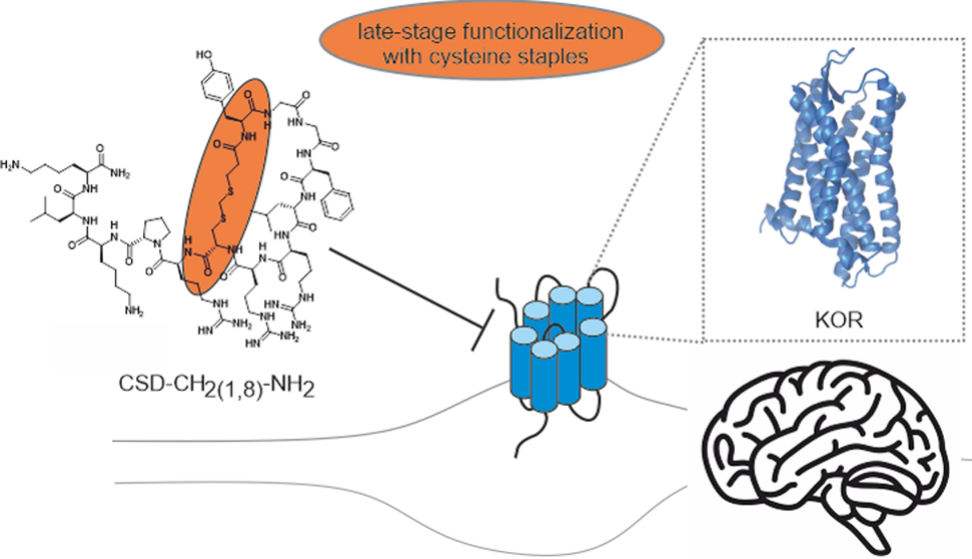

The κ-opioid receptor (KOR) is an attractive target for the development of novel drugs. KOR agonists are potentially safer pain medications, whereas KOR antagonists are promising drug candidates for the treatment of neuropsychiatric disorders. Hitherto, the vast majority of selective drug leads that have been developed for KOR are small molecules. In this study, novel peptide probes were designed by using an endogenous dynorphin A_1–13_ sequence as a template for peptide stapling via late-stage cysteine functionalization. Leveraging this strategy, we developed a stable and potent KOR antagonist, CSD-CH_2(1,8)_-NH_2_, with approximately 1000-fold improved selectivity for KOR over μ- and δ-opioid receptors. Its potent competitive KOR antagonism was verified in KOR-expressing cells, peripheral dorsal root ganglion neurons, and using the tail-flick and rotarod tests in mice. This work highlights the value of cysteine stapling to develop selective peptide probes to modulate central KOR function, as innovative peptide drug candidates for the treatment of KOR-related illnesses.

## Introduction

The wide expression of the κ-opioid receptor (KOR) throughout the central nervous system (CNS) and the peripheral nervous system (PNS) and its involvement in the control of important physiological processes including antinociception, motivation, emotion, and cognition have stimulated the development of KOR ligands for the treatment of pain, itch, and neuropsychiatric disorders.^1,2^ KOR agonists provide promising potential to develop safer and more effective analgesics since they lack the undesired effects typical for μ- (MOR) and δ- (DOR) opioid receptor agonists, including respiratory depression, constipation, tolerance, or addiction.^3^ Nevertheless, opioid-mediated activation of KOR is often associated with sedation, dysphoria, and hallucinations, which have precluded the clinical development of KOR agonists.^4,5^ Over recent years, biased opioid ligands selectively activating the G protein over β-arrestin signaling pathways have provided an avenue to develop therapeutics with reduced KOR side effects.^6^ However, it remains uncertain whether biased agonism offers therapeutic benefits.^7 –10^

In addition to pain-relieving KOR agonists, there is considerable interest in developing selective KOR therapeutic agents for the treatment of neuropsychiatric disorders.^1^ Notably, KOR antagonists have demonstrated the potential to relieve symptoms of depression, anxiety, or drug abuse.^1^ The vast majority of these ligands are small molecules, and only a few peptidic KOR antagonists have been developed to date.^1,11^ Dynantin is a potent and selective peptide KOR antagonist with antidepressant and anxiolytic-like therapeutic potential whose plasma stability and permeability to enter the CNS have been greatly improved by using a glyco-liposome delivery system.^12,13^ Hence, expanding the repertoire of peptide KOR antagonists as pharmacological tools to interrogate KOR signaling and potentially therapeutic candidates is of significant interest.

Dynorphin (dyn) A_1–17_ is the endogenous peptide KOR ligand that plays a pivotal role in a plethora of physiological functions.^14^ It comprises an N-terminal “message” motif (YGGF), common to all endogenous opioid peptides, and a C-terminal “address” sequence (LRRIRPKLKWDNQ) required for binding and activation of KOR.^15^ Structure–activity relationship studies of dynA_1–17_ have revealed residues and motifs essential for its pharmacological activity at KOR.^16^ However, dynA_1–17_ does not have “drug-like” properties: (i) it is linear and flexible and can adopt multiple conformational states, which may explain why it displays significant affinity toward MOR and DOR,^17,18^ (ii) dynA fragments are able to signal via all the three opioid receptors,^18,19^ and (iii) it has a short half-life and susceptibility to metabolic degradation—common to linear peptides in general.^20^ To leverage dynA as a template for the KOR ligand, designing a range of chemical strategies, including cyclization, N- or C-terminal modification, incorporation of non-canonical and d-amino acids, and peptide backbone modifications, is required.^9,20,21^

More generally, constrained peptides have evolved into a promising class of next-generation therapeutics.^22^ Many such constrained peptides have either been approved or are entering late-stage clinical trials for a range of therapeutic applications.^21^ Peptide stapling using cysteine crosslinking has emerged as an attractive approach to induce conformational restraint of peptides. Linear peptides comprise a high degree of conformational flexibility; hence, they may exist in high entropy states and sample a variety of conformations in solution.^23^ By contrast, conformational restriction reduces the flexibility of peptides and thus minimizes the entropy loss upon binding, which often results in enhanced stability and membrane permeability as well as increased affinity and selectivity of stapled peptides to their target proteins.^23−25^

In this study, we applied cysteine stapling to design selective cysteine-stapled dynA_1–13_ (CSD) analogues with desired pharmacological properties. Employing this strategy, we were able to develop a stable KOR-specific competitive antagonist CSD-CH_2(1,8)-_NH_2_ with nanomolar affinity. Our findings suggest that this novel ligand is a valuable research tool to study KOR-dependent calcium mobilization in peripheral neurons and antagonize centrally mediated effects of the KOR small-molecule agonist U50,488 in mice. Cysteine stapling holds the potential to design selective and stable peptide ligands with desired pharmacological properties to interrogate KOR pharmacology and develop therapeutic leads for KOR-mediated disorders.

## Results

### Design and Synthesis of CSD-OH Analogues

**Scheme 1:**
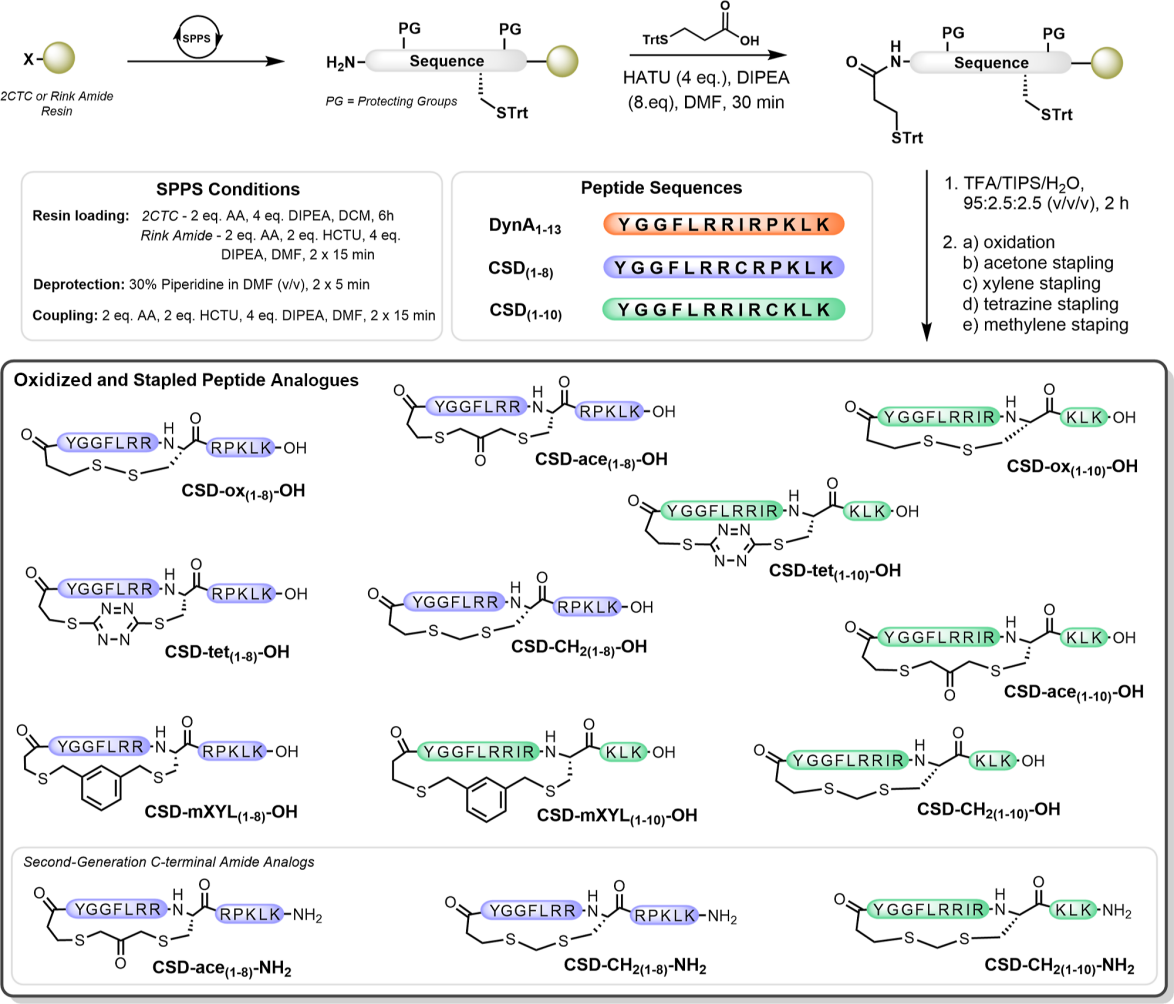
Overview of the Synthetic Strategy Used to Generate Oxidized and Stapled Dynorphin Analogues 2CTC: 2-chlorotrityl chloride; HATU: 2-(7-aza-1*H*-benzotriazole-1-yl)-1,1,3,3-tetramethyluronium hexafluorophosphate; HCTU: 2-(6-chloro-1*H*-benzotriazol-1-yl)-1,1,3,3-tetramethylaminium hexafluorophosphate; DIPEA: *N*,*N*-diisopropylethylamine; TFA: trifluoroacetic acid; TIPS: triisopropylsilane. For details of synthesis conditions, please refer to the Materials section.

To design constrained dynA ligands to target KOR, we aimed to utilize a strategy that could be implemented at a late stage, on unprotected peptide scaffolds, and that would be amenable to diversification. Lactam bridges, disulfide linkages, and carbon–carbon crosslinks have previously been explored to constrain dynA analogues with varying success.^26−30^ However, these strategies do not provide synthetic flexibility to rapidly diversify analogues with varying crosslinker functionality. Cysteine staples have emerged as a late-stage functionalization strategy that can be used to modulate G protein-coupled receptor (GPCR) pharmacology^31^ but hitherto have not been investigated in the design of KOR ligands. We sought to apply a cysteine stapling strategy to constrain a dynA_1–13_ peptide template bearing a native C-terminal carboxylic acid.^32^ A cysteine residue was introduced by substituting either isoleucine at position 8 or proline at position 10, and a second thiol was manually coupled onto the N-terminus using 3-(tritylthio)propionic acid. This generated two scaffolds (CSD_(1–8)_ and CSD_(1–10)_) that could undergo macrocyclization via disulfide formation or using cysteine stapling strategies (Scheme 1). Four cysteine stapling strategies were used to modify the unprotected peptide scaffolds, leading to the formation of methylene (CH_2_)-, acetone (ace)-, *m*-xylene (*m*XYL)-, and tetrazine (tet)-stapled motifs, to go alongside disulfide bond-constrained (ox) analogues. Overall, this strategy yielded 10 cysteine-stapled dynA_1–13_ analogues with diverse crosslinkers, which we named cysteine-stapled dyn (CSD) analogues.

### Pharmacological Screening of CSD-OH Analogues

**Figure 1:**
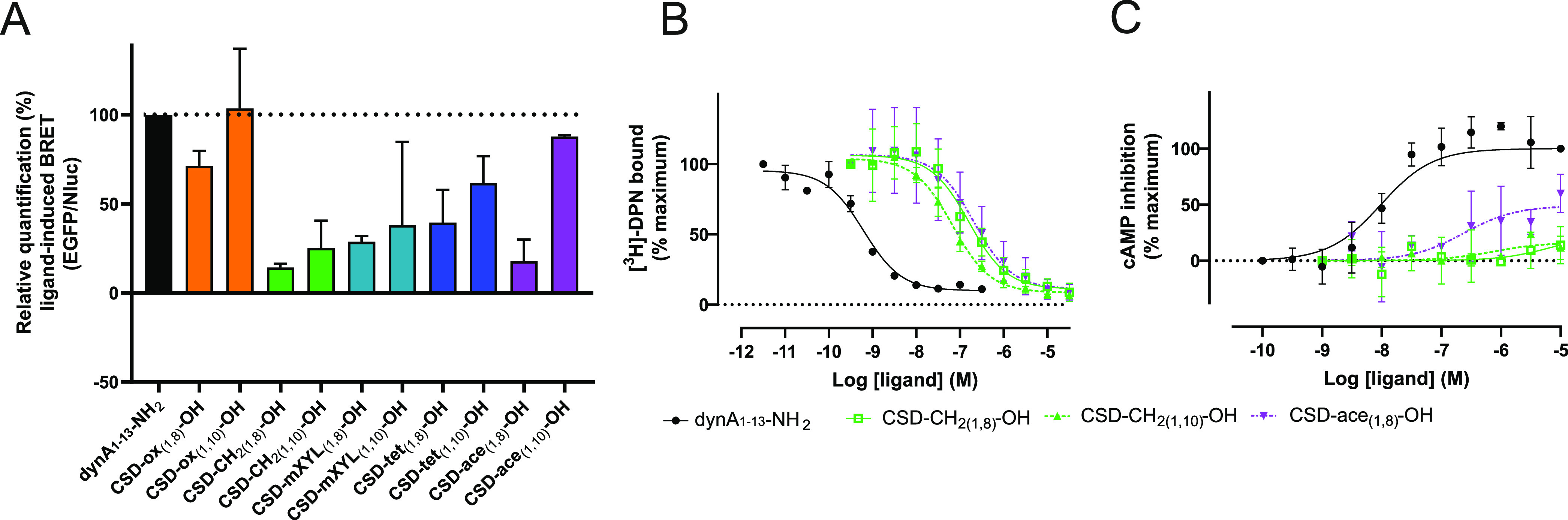
Receptor pharmacology of CSD analogues with C-terminal carboxylic acid at KOR. (A) Ligands at a concentration of 10 μM were added in the BRET assay following the establishment of the baseline for 5 min with furimazine (Nluc substrate). CSD-_ox(1,8)_-OH (orange bar), CSD-_ox(1,10)_-OH (orange bar), CSD-CH_2(1,8)_-OH (green bar), CSD-CH_2(1,10)_-OH (green bar), CSD-*m*XYL_(1,8)_-OH light blue bar), CSD-*m*XYL_(1,10)_-OH (light blue bar), CSD-tet_(1,8)_-OH (dark blue bar), CSD-_tet(1,10)_-OH (dark blue bar), CSD-ace_(1,8)_-OH (violet bar), and CSD-ace_(1,10)_-OH (violet bar) were subsequently measured for 30 min. DynA_1–13_-NH_2_ (10 μM) was used as a positive control. Data are from three independent experiments and are shown as mean ± SD. (B) Radioligand binding assay was carried out using 1 nM [^3^H]-diprenorphine (DPN) in HEK293 cell membrane preparations stably expressing mouse KOR. Cells were treated with indicated concentrations of CSD-CH_2(1,8)_-OH (*n* = 3, solid green line, open green squares), CSD-CH_2(1,10)_-OH (*n* = 3, dashed green line, green triangles), CSD-ace_(1,8)_-OH (*n* = 2, dashed violet line, inverted violet triangles), and dynA_1–13_-NH_2_ (*n* = 3, solid black line, black circles). The specific binding was calculated by subtracting the total from the non-specific binding. Data are presented as mean ± SD and are normalized to the percentage of maximum binding of dynA_1–13_-NH_2_. (C) KOR activation upon treatment with CSD-CH_2(1,8)_-OH (*n* = 3, solid green line, open green squares), CSD-CH_2(1,10)_-OH (*n* = 3, dashed green line, green triangles), and CSD-ace_(1,8)_-OH (*n* = 2, dashed violet line, inverted violet triangles) was concentration dependently measured by quantifying cAMP in HEK293 cells stably expressing mouse κ-opioid receptor (KOR). DynA_1–13_-NH_2_ (black line and circles) was used as a positive control (*n* = 3). Data represent mean ± SD and are normalized to the percentage of maximum activation of dynA_1–13_-NH_2_ detected at the highest concentration.

To identify novel peptide KOR ligands (antagonists, partial and full agonists, as well as biased molecules), we screened 10 initially designed peptides and explored their ability to recruit β-arrestin-2. For this, a kinetic BRET experiment was used with HEK293 cells transiently expressing mouse KOR coupled to EGFP and β-arrestin-2 fused to NanoLuc (Nluc). The data demonstrated that dynA_1–13_ analogues with C-terminal carboxylic acid recruit β-arrestin-2, yet with distinct levels of BRET efficiency when using a saturating concentration of 10 μM (Figures 1A and S2, Supporting Information). For instance, compared to dynA_1–13_-NH_2_, CSD-CH_2(1,8)_-OH, CSD-CH_2(1,10)_-OH, and CSD-ace_(1,8)_-OH exhibited only minor β-arrestin-2 recruitment with efficiencies less than 25% (Figures 1A and S2, Supporting Information). By contrast, CSD-ox_(1,8)_-OH and CSD-ace_(1,10)_-OH recruited β-arrestin-2 to an extent similar to dynA_1–13_-NH_2_ (Figures 1 and S2, Supporting Information). Since CSD-CH_2(1,8)_-OH, CSD-CH_2(1,10)_-OH, and CSD-ace_(1,8)_-OH induced only minimal or no β-arrestin-2 recruitment, we next conducted radioligand binding studies to examine the KOR affinity. All ligands displayed KOR affinity in the nanomolar concentration range (Figure 1B and Table 1). CSD-CH_2(1,8)_-OH, CSD-CH_2(1,10)_-OH, and CSD-ace_(1,8)_-OH exhibited *K*_i_ values of 77, 31, and 91 nM, respectively (Figure 1B and Table 1). In addition to radioligand binding measurements, we tested their ability for KOR-mediated inhibition of adenylyl cyclase-dependent cAMP formation. In the functional cAMP assay, CSD-CH_2(1,8)_-OH and CSD-CH_2(1,10)_-OH were inactive up to concentrations of 10 μM, suggesting potential antagonism of these ligands at KOR, whereas CSD-ace_(1,8)_-OH partially activated KOR with an EC_50_ of 270 nM and an *E*_max_ of 57% (Figure 1C and Table 1).

**Table 1:** Pharmacological Properties of CSD Peptide Leads at KOR^a^

ligand	potency/efficacy cAMP		affinity
	EC_50_ ± SD (M)	*E*_max_ ± SD (%)	*K*_i_ ± SD (M)
dynA_1–13_-NH_2_	9.1 ± 1.6 × 10^–9^	100	2.9 ± 0.4 × 10^–10^
CSD-CH_2(1,8)_-OH	n.a.	n.a.	7.7 ± 1.9 × 10^–8^
CSD-CH_2(1,10)_-OH	n.a.	n.a.	3.1 ± 0.6 × 10^–8^
CSD-ace_(1,8)_-OH	2.7 ± 0.8 × 10^–7^	57 ± 11	9.1 ± 3.5 × 10^–8^
CSD-CH_2(1,8)_-NH_2_	n.a.^b^	n.a.	6.8 ± 1.8 × 10^–9^
CSD-CH_2(1,10)_-NH_2_	5.4 ± 1.5 × 10^–7^	49 ± 15	5.6 ± 1.3 × 10^–9^
CSD-ace_(1,8)_-NH_2_	7.4 ± 1.8 × 10^–7^	73 ± 14	3.2 ± 1.3 × 10^–8^

a Data are from two to three independent experiments; dyn, dynorphin; n.a., not active.

b Functional affinity of 32 nM was derived from the Schild regression analysis.

### Pharmacological Properties of C-Terminally Amidated CSD Ligands at KOR

**Figure 2:**
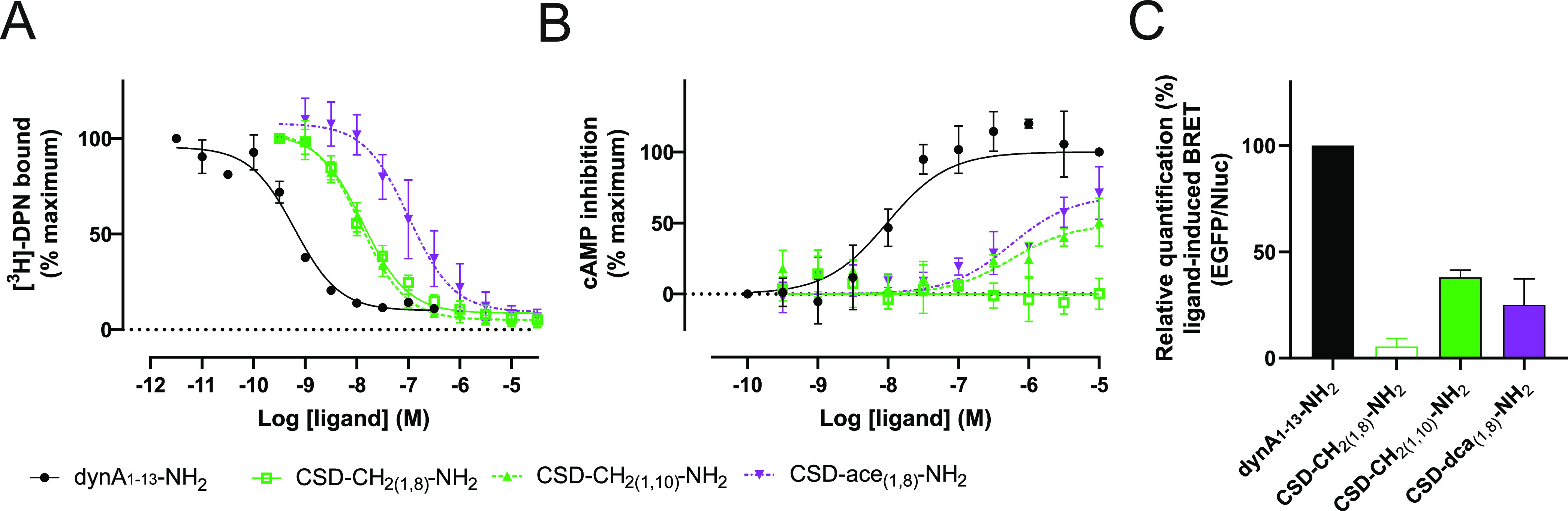
Receptor pharmacology of C-terminally amidated CSD ligands at KOR. (A) Affinity of CSD-CH_2(1,8)_-NH_2_ (*n* = 3, solid green line, open green squares), CSD-CH_2(1,10)_-NH_2_ (*n* = 3, dashed green line, green triangles), and CSD-ace_(1,8)_-NH_2_ (*n* = 3, dashed violet line, inverted violet triangles) was measured in radioligand binding studies on HEK293 cell membranes stably expressing mouse KOR. Specific binding was obtained via subtraction of the total from the non-specific binding. Graph of dynA_1–13_-NH_2_ (black solid line, black circles) is included for comparison. Data are presented as mean ± SD and are normalized to the percentage of the maximum binding detected in the presence of 1 nM [^3^H]-diprenorphine (DPN) and the absence of competing ligands. (B) G_αi_-mediated cAMP inhibition of mouse KOR was measured in HEK293 cells stably expressing mouse κ-opioid receptor (KOR) after the cell treatment with CSD-CH_2(1,8)_-NH_2_ (*n* = 3, solid green line, open green squares), CSD-CH_2(1,10)_-NH_2_ (dashed green line, green triangles), and CSD-ace_(1,8)_-NH_2_ (dashed violet line, inverted violet triangles). DynA_1–13_-NH_2_ was used as a positive control (*n* = 3, solid black line, black circles, same as in Figure 1). Data are shown as mean ± SD. Normalization of data was performed according to the percentage of the maximum activation of dynA_1–13_-NH_2_. (C) Bioluminescence resonance energy transfer (BRET) assay was employed to measure the transient interaction of κ-opioid receptor (KOR) with β-arrestin-2 in HEK293 cells over time following cell treatment with 10 μM CSD-CH_2(1,8)_-NH_2_ (*n* = 3, open green bar), CSD-CH_2(1,10)_-NH_2_ (*n* = 3, green bar), and CSD-ace_(1,8)_-NH_2_ (*n* = 3, violet bar). DynA_1–13_-NH_2_ (black filled bar) was used a positive control (*n* = 3, black bar) and furimazine as a substrate for luciferase which was incubated for 5 min prior to the establishment of baseline and ligand treatment. DynA_1-13_ control is identical in Figures 1B,C and 2A,B.

Previous studies reported that the C-terminal amidation of dynA-based ligands contributes to their affinity at KOR.^15,16^ Accordingly, we reasoned that introducing a C-terminal amidation to the most promising peptides, i.e., CSD-CH_2(1,8)_-OH, CSD-CH_2(1,10)_-OH, and CSD-ace_(1,8)_-OH, could further improve their desired pharmacological properties at KOR. Peptides containing an amidated C-terminus were synthesized analogous to peptides with the carboxyl group at the C-terminus (Scheme 1). Following synthesis, the peptides were subject to pharmacological characterization. We first assessed the affinity of C-terminally amidated peptides in radioligand binding studies. The affinity of CSD-CH_2(1,8)_-NH_2_ (*K*_i_ = 7 nM), CSD-CH_2(1,10)_-NH_2_ (6 nM), and CSD-ace_(1,8)_-NH_2_ (32 nM) improved by approximately 12-, 6-, and 3-fold, respectively (Figure 2A and Table 1). We then conducted cAMP assays to explore the potency and efficacy of newly synthesized ligands to modulate G protein signaling. Intriguingly, compared to their counterparts with a carboxyl group at the C-terminus, the C-terminal-amidated peptides displayed disparate potencies and efficacies (Figure 2B and Table 1). CSD-CH_2(1,8)_-NH_2_ was inactive at KOR on its own compared to CSD-CH_2(1,10)_-NH_2_, which acted as a partial agonist following C-terminal amidation with an EC_50_ and *E*_max_ of 540 nM and 49%, respectively (Figure 2B and Table 1). On the other hand, the C-terminal amidation of CSD-ace_(1,8)_-NH_2_ decreased its potency to EC_50_ = 740 nM (Figure 2B and Table 1). Finally, we screened peptides in the kinetic β-arrestin-2 recruitment assay. Like previously characterized analogues, all the three peptides did not or only partially recruit β-arrestin-2 (ligand induced BRET CSD-CH_2(1,8)_-NH_2_: 5%, CSD-CH_2(1,10)_-NH_2_: 38%, CSD-ace_(1,8)_-NH_2_: 25%, as compared to dynA_1-13_-NH_2_: 100%) at concentrations of 10 μM (Figures 2C and S2A–C, Supporting Information).

### Increased Selectivity and Stability of KOR CSD Ligands

**Figure 3:**
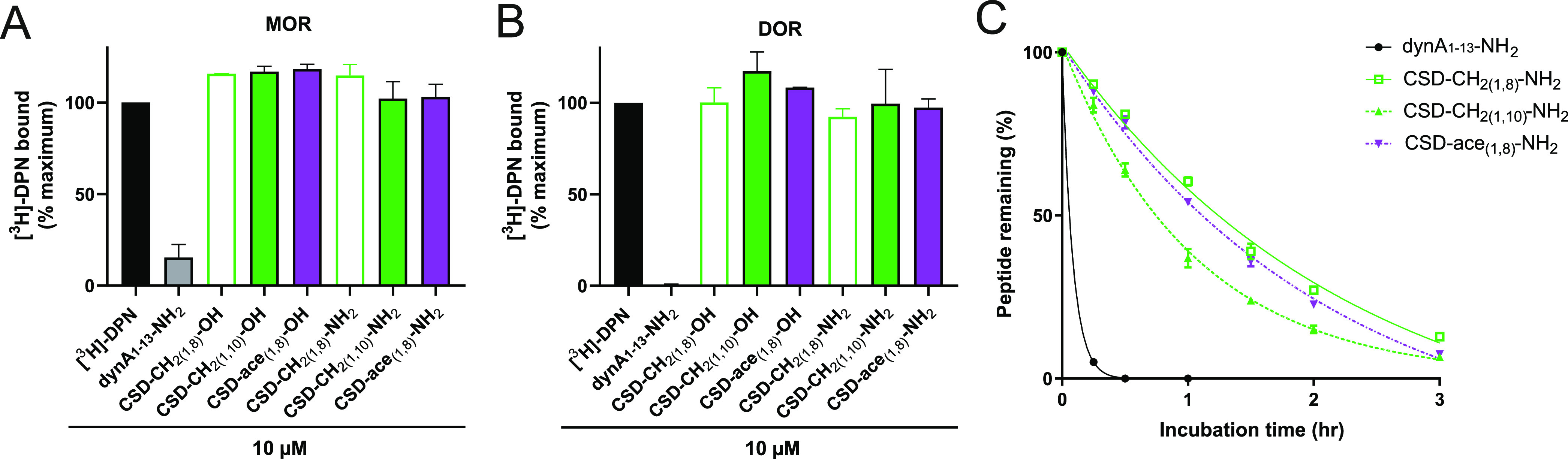
Subtype selectivity and stability of CSD-NH_2_ peptides. CSD-NH_2_ peptide ligands were measured for displacing 1 nM tritiated diprenorphine ([^3^H]-DPN, black bar) in radioligand binding studies in HEK293 cells stably expressing (A) mouse μ-opioid receptor (MOR) and (B) mouse δ-opioid receptor (DOR) (*n* = 2). DynA_1–13_-NH_2_ (black bars) served as a positive control (10 μM, *n* = 2). To determine specific binding, the total from non-specific binding was subtracted. CSD-CH_2(1,8)_ peptides are shown in green, empty bars, CSD-CH_2(1,10)_-NH_2_ ligands in green bars, and CSD-ace_(1,8)_-NH_2_ peptides are displayed in violet bars. (C) Stability of dynA_1–13_-NH_2_ (*n* = 3, solid black line, black circles), CSD-CH_2(1,8)_-NH_2_ (*n* = 3, green solid line, green empty squares), CSD-CH_2(1,10)_-NH_2_ (*n* = 3, green dashed line, green triangles), and CSD-ace_(1,8)_-NH_2_ (violet dashed line, violet inverted triangles) was measured in human plasma.

We next examined the selectivity profile of the three peptides comprising NH_2_ at the C-terminus by measuring their binding affinity in HEK293 cell membrane preparations stably expressing mouse MOR and DOR. We tested a concentration of 10 μM for each peptide ligand and demonstrated that all the three peptides are KOR selective as they did not significantly displace the radioligand from MOR and DOR at this concentration (Figure 3A,B). Similar findings were observed for the three peptides with a C-terminal COOH (Figure S3, Supporting Information). It has been reported that cysteine stapling improves the stability of peptides.^23,24^ To probe this suggestion, we first measured the stability of three peptides with the C-terminal COOH in human serum by reversed-phase high-performance liquid chromatography (RP-HPLC). Interestingly, these peptides degraded within 15 min, similar to the native dynA_1–13_-NH_2_ (data not shown). Since C-terminal amidation improved the binding affinity of CSD-CH_2(1,8)_-NH_2_, CSD-CH_2(1,10)_-NH_2_, and CSD-ace_(1,8)_-NH_2_, we then determined the stability of these peptides in human serum by RP-HPLC. In fact, C-terminal amidation of CSD peptides improved their stability compared to the linear dyn A_1–13_-NH_2_ (*t*_1/2_ = 4 min) with CSD-CH_2(1,8)_-NH_2_ (*t*_1/2_ = 95 min) being the most stable peptide ligand (Figure 3C).

### Competitive Antagonism of CSD_(1,8)_-NH_2_ and Its Inhibitory Action on Calcium Mobilization in DRGs

**Figure 4:**
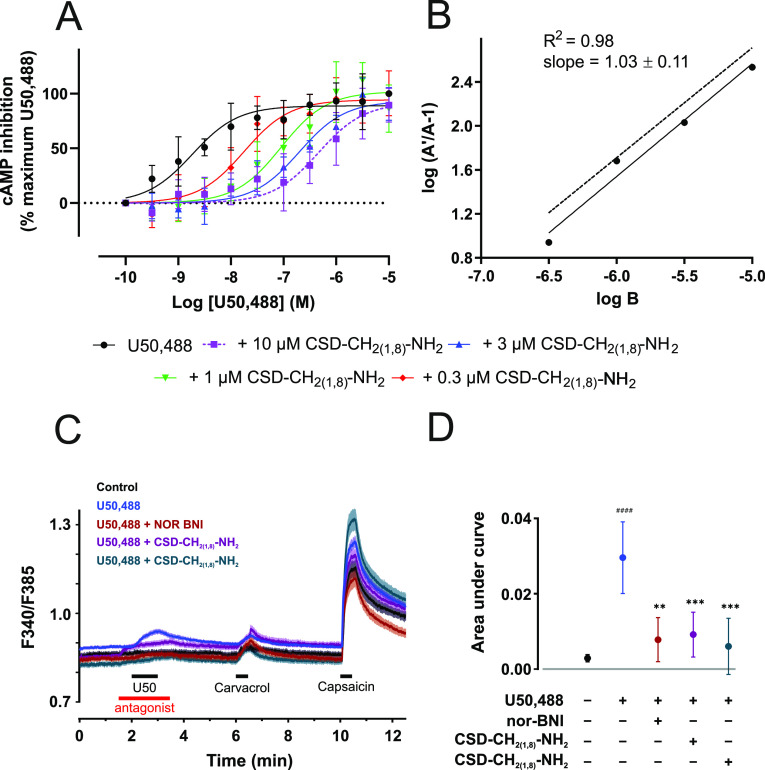
Competitive antagonism of CSD-CH_2(1,8)_-NH_2_ at KOR and its inhibition of calcium mobilization in DRGs. (A) Concentration–response curves of U50,488 (solid black line, black circles) in the absence and presence of 0.3 μM (solid red line, red diamonds), 1 μM (solid green line, green inverted triangles), 3 μM (solid blue line, blue triangles), and 10 μM (dashed violet line, violet squares) CSD-CH_2(1,8)_-NH_2_. G_αi_-mediated cAMP inhibition of mouse KOR was measured in HEK293 cells stably expressing mouse κ-opioid receptor (KOR). Data were normalized to the percentage of maximum activation of U50,488 (*n* = 4). (B) Schild regression analysis. *A* denotes the EC_50_ of U50,488 in the presence of CSD-CH_2(1,8)_-NH_2_, *A*′ is the EC_50_ of U50,488, and *B* indicates the logarithm of concentration of CSD-CH_2(1,8)_-NH_2_. The dotted line with a slope of 1 (*n* = 4) is used for comparison. (C) Mean traces of calcium signals in dorsal root ganglion (DRG) cultures without stimulation (Control, black traces, *n* = 648) or stimulated by U50,488 (U50 10 μM, blue traces, *n* = 1467), U50,488 (10 μM) in combination with nor-BNI (0.5 μM, red traces, *n* = 537), U50,488 (10 μM) in combination with CSD-CH_2(1,8)_-NH_2_ (1 μM, violet traces, *n* = 1126), and U50,488 (10 μM) in combination with CSD-CH_2(1,8)_-NH_2_ (10 μM, cyan traces, *n* = 720). The antagonists nor-BNI and CSD-CH_2(1,8)_-NH_2_ were pre-applied, followed by addition of the agonist. Carvacrol (100 μM) and capsaicin (1 μM) served as controls for TRPA1 and TRPV1, respectively. Control was an external solution. A potassium chloride (KCl) solution (60 mM) added at the end of each recording was used as a positive control for detecting viable cells and generated similar response amplitudes. Lines represent the mean, and the shaded areas represent the standard error of the mean. (D) Quantification by area under the curve (AUC) of control (Control, black), U50,488 (U50, 10 μM, blue), nor-BNI (0.5 μM, red), U50 (10 μM) in combination with CSD-CH_2(1,8)_-NH_2_ (1 μM, violet), and U50 (10 μM) in combination with CSD-CH_2(1,8)_-NH_2_ (10 μM, cyan) for the interval 120–180 s. Data are shown as mean ± 95% C.I. from two independent experimental days. One-way ANOVA with Tukey’s multiple comparisons test. Two asterisks indicate the significance between U50,488 (10 μM) and U50,488 (10 μM) + nor-BNI (0.5 μM), while three asterisks represent the significance between U50,488 (10 μM) and U50,488 (10 μM) + CSD-CH_2(1,8)_-NH_2_ (1 μM) as well as U50,488 (10 μM) and U50 + CSD-CH_2(1,8)_-NH_2_ (10 μM) (***p < 0.001). Four hashtags indicate the significance between U50,488 (10 μM) and Control (^####^*p* < 0.0001).

To determine the mechanism of antagonism of CSD-CH_2(1,8)_-NH_2_, we measured adenylyl cyclase-mediated cAMP inhibition at KOR using the Schild regression analysis. KOR expressed in HEK293 cells was activated by the small-molecule agonist U50,488 in the absence and presence of increasing concentrations of CSD-CH_2(1,8)_-NH_2_. We observed a rightward shift of the concentration–response curves of U50,488, with no change in *E*_max_ typical for competitive antagonism (Figure 4A). Detailed Schild analysis of CSD-CH_2(1,8)_-NH_2_ exhibited a linear regression slope of 1.03 and a pA2 value of 7.5, which corresponds to an average functional affinity of 32 nM, thus demonstrating the competitive mechanism of the CSD-CH_2(1,8)_-NH_2_ antagonist (Figure 4B). Importantly, we verified the antagonistic activity of CSD-CH_2(1,8)_-NH_2_ in a physiological context by exploring its ability to modulate calcium mobilization in peripheral DRGs known to express KOR.^33−35^ Using single-cell calcium microfluorimetry on neuronal populations described by their response to the TRPA1 agonist carvacrol and the TRPV1 agonist capsaicin, we applied CSD-CH_2(1,8)_-NH_2_ in combination with the KOR agonist U50,488 (Figure 4C,D). To confirm antagonism of CSD-CH_2(1,8)_-NH_2_, we co-applied it with U50,488 and observed that CSD-CH_2(1,8)_-NH_2_ significantly blocks the action of U50,488 (Figure 4C,D). This effect was further supported by the KOR-selective antagonist norbinaltorphimine (nor-BNI) which inhibited the activity of U50,488 (Figure 4C,D). Taken together, these findings suggest that CSD-CH_2(1,8)_-NH_2_ inhibits the action of U50,488 in both KOR-transfected HEK293 cells and DRG neurons, which endogenously express KOR.

### In Vivo KOR Antagonism of CSD_(1,8)_-NH_2_ after s.c. Administration in Mice

**Figure 5:**
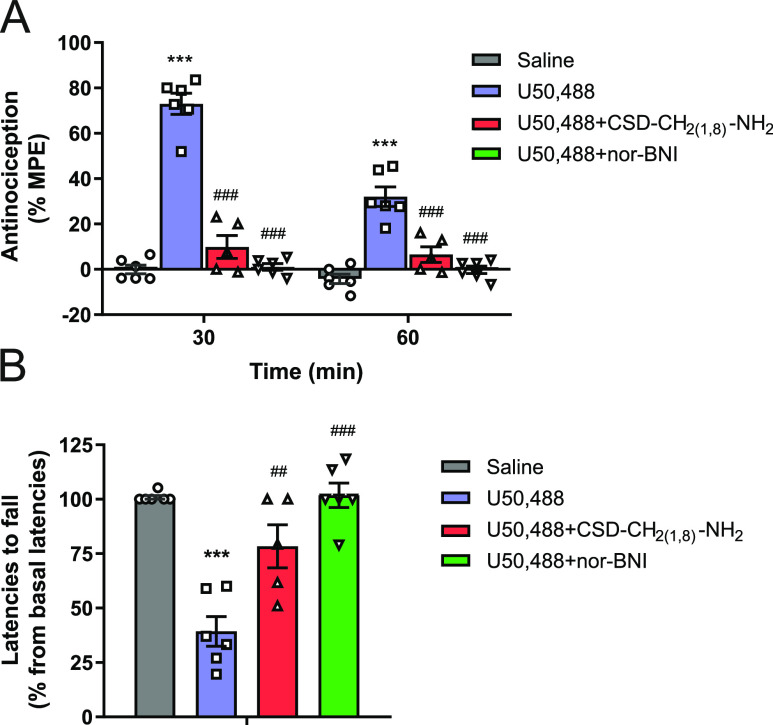
In vivo KOR antagonist activity and site of action of CSD-CH_2(1,8)_-NH_2_. (A) Antagonism of U50,488-induced antinociception by CSD-CH_2(1,8)_-NH_2_ and nor-BNI after s.c. administration in the radiant heat tail-flick assay in mice. (B) Antagonism by CSD-CH_2(1,8)_-NH_2_ and nor-BNI on the effect of U50,488 in the rotarod test in mice after s.c. administration. Groups of mice were s.c. treated with CSD-CH_2(1,8)_-NH_2_ (20 mg/kg) or nor-BNI (10 mg/kg), 15 min or 24 h, respectively, prior to administration of U50,488 (5 mg/kg), and (A) tail-flick latencies (at 30 and 60 min) or (B) latencies to fall from the rotarod (at 30 min) were determined. Values represent means ± SEM (*n* = 5–6 mice per group). ***P < 0.001 vs saline group and ^##^*P* < 0.01 and ^###^*P* < 0.001 vs U50,488-treated group, one-way ANOVA with Tukey’s post hoc test.

The in vivo KOR antagonist activity of CSD-CH_2(1,8)_-NH_2_ was evaluated in mice after subcutaneous (s.c.) administration by assessing the CSD-CH_2(1,8)_-NH_2_ effect on U50,488-induced antinociception in a mouse model of acute thermal nociception, the radiant heat tail-flick assay. U50,488 was used as a prototypical centrally acting KOR agonist. Pretreatment of mice with CSD-CH_2(1,8)_-NH_2_ (20 mg/kg) significantly antagonized the antinociceptive effect of U50,488 (Figure 5A). Similarly, s.c. administration of the standard KOR antagonist nor-BNI (10 mg/kg) also blocked the antinociceptive response of U50,488 (Figure 5A). Activation of central KOR is well recognized to induce the CNS effects, such as sedative effects that can be readily observed in animals by a marked decrease in the locomotor activity. As shown in Figure 5B, U50,488, in an antinociceptive dose of 5 mg/kg, significantly affected motor coordination in the rotarod test in mice after s.c. administration. To evaluate if CSD-CH_2(1,8)_-NH_2_ reverses the motor impairment caused by U50,488 through the central KOR, mice were pretreated with CSD-CH_2(1,8)_-NH_2_ (20 mg/kg) prior to U50,488. Administration of CSD-CH_2(1,8)_-NH_2_ to mice significantly antagonized the effect of U50,488 in the rotarod test, comparable to the effect produced by nor-BNI (Figure 5B). These behavioral data show that CSD-CH_2(1,8)_-NH_2_ has in vivo a KOR-mediated antagonist activity by modulating central KOR function.

## Discussion

KOR and its endogenous opioid peptide dynA mediate a diverse array of physiological actions including nociception, reward, and stress.^36^ These neurobiological functions allow potential utilization of KOR pharmacology for numerous therapeutic applications, ranging from KOR agonists as effective, non-addictive pain medications^6^ to KOR antagonists for neuropsychiatric and neurological disorders including depression, anxiety, or drug abuse.^1^ While there are many small molecules available as research tools or drugs, there are only a few peptide ligands of KOR that have advanced into the clinic or have become reliable chemical probes.^9,11,37,38^ The reasons are the intrinsic lack of stability and low membrane permeability of peptides, resulting in unfavorable pharmacokinetic properties.^39^ These limitations have fostered the development of technological innovations to advance the status of peptides with “drug-like” features.

Peptide stapling has been widely utilized to develop constrained peptides to improve their “drug-like” pharmacokinetic and pharmacodynamic properties.^23−25,40^ This strategy has been applied to design innovative ligands of other GPCRs, in particular galanin and neuropeptide Y receptors,^41^ orexin receptors,^42^ oxytocin and vasopressin receptors,^43^ and the melanocortin 1 receptor.^31^ Hitherto, cysteine stapling with late-stage functionalization moieties has only sparsely been employed for opioid receptors. To investigate its potential as underutilized handles in targeting opioid receptors, we designed KOR targeting dynA_1–13_ analogues constrained with diverse cysteine-stapled moieties including methylene, acetone, *m*-xylene, and tetrazine-stapled motifs. Interestingly, the cysteine-stapled dynA_1–13_ analogue CSD-CH_2(1,8)_-NH_2_ with a C-terminal amidation is a competitive KOR antagonist with nanomolar affinity and over 1000-fold enhanced selectivity for KOR over MOR and DOR. These findings are in line with previous studies reporting the need of an amidated C-terminus for increased affinity and selectivity of dynA analogues toward KOR.^16^ The extracellular loop 2 of KOR that is rich in negatively charged residues^17^ has been suggested to play an essential role in maintaining the high affinity and selectivity of dynA at KOR.^44−46^ This allows us to speculate that a C-terminal amide of peptide analogues may interact with the acidic residues in the extracellular loop of KOR, thereby leading to an increase in KOR affinity.

The development of selective peptide KOR antagonists using dynorphin as a design template has been challenging due to the high degree of sequence homology between KOR, MOR, and DOR binding pockets.^11^ Thus, the engineering of a competitive KOR antagonist CSD-CH_2(1,8)_-NH_2_ by cysteine stapling expands the currently limited toolbox of potent and selective peptide KOR antagonists. Importantly, the ability of CSD-CH_2(1,8)_-NH_2_ to retain inhibitory KOR activity in a DRG neuronal primary cell model emphasizes its applicability as a valuable molecular probe to study KOR signaling in an endogenous environment. We also demonstrated the in vivo KOR antagonism of CSD-CH_2(1,8)_-NH_2_ in mice after s.c. administration based on its ability to reverse antinociception and sedation/motor impairment produced by U50,488. Furthermore, CSD-CH_2(1,8)_-NH_2_ had in vivo a KOR-mediated antagonist activity by modulating KOR function in the CNS.

More generally, multiple synthetic strategies have been developed to improve the metabolic stability of KOR peptide ligands, including substitution of natural l-amino acids with d- or non-canonical amino acids, peptide backbone modifications, and cyclization.^28−30,47^ Generating a library of diverse dynA-based cysteine staples enabled us to identify a peptide ligand with enhanced stability. Our lead molecule CSD-CH_2(1,8)_-NH_2_ demonstrated improved stability toward proteolytic degradation in human serum, making it a promising starting point for the design of KOR targeting therapeutics. This is of particular significance given the innovation to develop stable and peripherally restricted peptide KOR analgesics as an alternative to centrally acting small-molecule opioid painkillers frequently associated with undesired effects. A recent study has demonstrated that grafting dynA epitopes onto a plant-derived, cyclic peptide scaffold is a successful strategy to design potent peptide ligands with strong peripheral KOR activity. Efficacy was verified in a mouse model of chronic visceral pain, and no signs of central effects were observed, considering motor coordination or sedation.^47^ On the other hand, the cyclic dynA analogue zyklophin is a potent antagonist of KOR following systemic administration in vivo.^48^ Accordingly, zyklophin is a good example of a stable peptide ligand with an advantage over small molecules: its effects have a shorter duration as compared to long-lasting small-molecule KOR antagonists.^48^ Hence, as an alternative to peptide cyclization, stapling peptides using cysteine crosslinking, like CSD-CH_2(1,8)_-NH_2_, are not only a powerful strategy to develop stable peptides as research tools for disentangling KOR pharmacology but also provide enticing opportunities to engineer KOR targeting peptides with a desired site of action, for instance, by improving their delivery into the CNS.

In conclusion, applying peptide cysteine stapling and the endogenous dynA as a molecular template, we developed a potent and stable KOR antagonist with superior KOR selectivity (approx. 3 orders of magnitude) over other opioid receptors. The ongoing opioid crisis underscores the necessity to develop novel pharmacological tools and therapeutics for pain treatment and several other disorders.^3^ Our study reinforces the potential of late-stage functionalization to design pharmacological probes and lead molecules with desired pharmacological characteristics. Such ligands may, in the future, become useful drug candidates for KOR-related indications.

## Experimental Section

### Materials

Dynorphin (dyn) A_1–13_ amide trifluoroacetate salt was purchased from Bachem (Austria). Naloxone, (±)-*trans*-U50,488 methanesulfonate salt, nor-BNI, carvacrol, and capsaicin were obtained from Sigma (Austria). [^3^H]-diprenorphine (DPN) was ordered from PerkinElmer (Austria). cAMP G_i_ kit was from Cisbio (France) and jetPRIME transfection reagent from Polyplus (Austria). Bromoacetic acid was ordered from Chem-Supply (Australia).

### Peptide Synthesis

Peptides were assembled using automated Fmoc solid-phase peptide synthesis (SPPS) using either rink amide or 2-chlorotrityl chloride resins on a 0.125 mmol scale. Amino acid couplings were performed with 2 equiv of amino acid, 2 equiv of HCTU, and 4 equiv of DIPEA in DMF (4 mL) for 15 min and repeated twice for each residue. Fmoc was deprotected using 30% piperidine in DMF for 5 min, repeated twice. CSD peptides were assembled using an N-terminal 3-(tritylthio)propanoic acid, manually coupled using 4 equiv of HATU and 8 equiv of DIPEA in 4 mL of DMF for 30 min. Each peptide was cleaved from the resin using a cleavage cocktail of TFA/TIPS/H_2_O (95:2.5:2.5) for 2 h, and the crude peptides were precipitated with cold Et_2_O. The precipitated peptides were then redissolved in H_2_O/MeCN (1:1) and lyophilized to yield a white powder. Each peptide was purified by reversed-phase high-performance liquid chromatography (RP-HPLC) on a Shimadzu Prominence HPLC system prior to oxidation and stapling.

Disulfide-bonded peptides (ox) were synthesized by stirring the reduced peptide precursor (5.2 μmol) in 50 mM ammonium bicarbonate (pH 8.5) at 1 mg/mL in an open reaction vessel. After 18 h, the mixture was acidified with TFA and purified by RP-HPLC.

Acetone (ace)-stapled peptides were prepared by dissolving the reduced peptide precursor (6.5 μmol) in 15 mL of 50 mM ammonium bicarbonate (pH 9.5) with 1 equiv of TCEP. 1,3-Dichloroacetone was (2 equiv) added from a 40 mM stock solution in MeCN. After stirring for 2 h, the reaction was acidified with TFA and purified by RP-HPLC.

*m*XYL-stapled analogues were prepared by dissolving the reduced peptide precursor (5.2 μmol) in 8 mL of 50 mM ammonium bicarbonate (pH 9.5) with 20% MeCN and 1 equiv of TCEP. 1,3-Bis(bromomethyl)benzene (1.5 equiv) was added dropwise from a 40 mM stock solution in DMF and mixed for 30 min before acidification with TFA and purification by RP-HPLC.

Tetrazine (tet)-stapled peptides were prepared by dissolving the reduced peptide precursor (6.2 μmol) in 12 mL of 50 mM ammonium bicarbonate (pH 5.5). The mixture was added to a 50 mL falcon tube containing 3 equiv of 3,6-dichloro-1,2,4,5-tetrazine in 12 mL of CHCl_3_ and vortexed for 5 min. The organic and aqueous layers were separated by centrifugation, and the aqueous layer was isolated. The organic layer was further washed with 50 mM ammonium bicarbonate, and aqueous isolates were combined, acidified by TFA, and purified by RP-HPLC.

Methylene (CH_2_)-stapled peptides were prepared by dissolving the reduced peptide precursor (6.2 μmol) in 12 mL of H_2_O/THF (5:1, v/v) with 1.5 equiv of K_2_CO_3_, 3 equiv of TCEP, 10 equiv of triethylamine, and 8 equiv of diiodomethane added sequentially. The mixture was stirred for 6 h before acidification with TFA and purification by RP-HPLC.

### Peptide Purification

Peptides were purified by RP-HPLC on preparative or semipreparative Phenomenex (Germany) Jupiter C_18_ columns (5 μm, 300 Å, 250 × 21.2 mm or 250 × 10 mm) applying a linear gradient from 5 to 65% solvent B (90% MeCN, 10% H_2_O, 0.05% TFA) and flow rates of 8 or 3 mL/min, respectively. Automatically collected fractions were analyzed by electrospray ionization mass spectrometry (ESI-MS) using a Shimadzu LCMS 2010 system and analytical RP-HPLC on a Phenomenex Jupiter C_18_ column (5 μm, 300 Å, 150 × 2 mm). The mass and purity of all peptides were confirmed by matrix-assisted laser desorption/ionization (MALDI) time-of-flight (TOF) MS using an AB Sciex 5800 spectrometer and analytical RP-HPLC using a Shimadzu UPLC system (Figures S4–S10 and Table S1, Supporting Information). MALDI-TOF MS was performed by mixing 3 μL of α-cyano-hydroxycinnamic acid (CHCA) and 0.5 μL of peptide and spotting 0.5 μL of the mixture onto the MALDI target plate. All peptides were purified to >95% purity.

### NMR

NMR was performed using a Bruker Avance III 600 MHz spectrometer equipped with a cryogenically cooled probe. Each peptide was prepared in a ∼2 mM solution of H_2_O/D_2_O (9:1, v/v), and experiments were run at 298 K with referencing to H_2_O at 4.70 ppm. ^1^H, TOCSY, and NOESY experiments were run for each sample. ^1^H NMR spectra are available in the Supporting Information (Figures S11–S23, Supporting Information).

### Stability Assay

The stability of peptide ligands was determined with human serum from male AB plasma (Sigma) centrifuged at 13,300*g* for 10 min to remove the lipid content. The supernatant was preincubated at 37 °C for 5 min before 10 μL of a peptide stock solution (3 mg/mL in H_2_O) was added to 300 μL of serum. A reference solution was prepared in phosphate-buffered saline (PBS) buffer. The serum was incubated at 37 °C and analyzed over a time course of 5 h with 40 μL of aliquots removed at time points *T* = 0, 15, 30, 60, 90, 120, 240, and 300 min. Each aliquot was quenched with 40 μL of 5 M urea and incubated on ice for 10 min. The proteins were then precipitated with the addition of 40 μL of 20% (w/v) trichloroacetic acid and further incubated on ice for 10 min. The samples were centrifuged for 10 min at 15,000*g*, and the supernatant was analyzed by analytical RP-HPLC using a Phenomenex Jupiter C_18_ column (5 μm, 300 Å, 150 × 2 mm) using a linear gradient from 0 to 50% solvent B (90% acetonitrile, 10% H_2_O, and 0.05% trifluoroacetic acid) and a flow rate of 1 mL/min. The percentage of peptide remaining was determined by the integration of the analyte in comparison to *T* = 0 min. The peptides were analyzed in triplicate, and data were fitted to an exponential decay curve using GraphPad Prism software.

### Cell Culture and Transfection

HEK293 cells were grown at 37 °C and cultured in Dulbecco’s modified Eagle’s medium (DMEM) supplemented with 10% fetal bovine serum and 50 U/mL penicillin as well as streptomycin. Cell transfection was performed with the jetPRIME transfection reagent as per the manufacturer’s protocol (Polyplus, France) using 2 μg of plasmids encoding mouse KOR-EGFP and/or human β-arrestin-2-nano luciferase. Cloning of mouse KOR into pEGFP-N1 vector and generation of a HEK293 cell line stably expressing mouse KOR-EFGP were performed as previously described.^47^

### Radioligand Displacement Binding Assays

HEK293 cell membranes stably expressing KOR, MOR, and DOR were prepared as described in Muratspahić et al.^47^ The dissociation constant *K*_d_ values for the mouse KOR were previously determined in a saturation binding assay.^49^ Non-specific binding was examined in the presence of 10 μM naloxone. Displacement binding was performed in a final volume of 300 μL containing [^3^H]-diprenorphine (1 nM final), peptide solution (4×), membranes (3.5–7 μg/assay), and standard binding buffer (50 mM Tris–HCl (pH 7.4), 10 mM MgCl_2_, and 0.1% BSA). Equilibrium was achieved by incubating displacement binding reactions at 37 °C for 1 h. The Skatron cell harvester device was utilized to terminate reactions by rapid filtration onto GF/C filters pre-soaked with 0.1% polyethylenimine. Displacement binding for MOR and DOR was conducted with 50–100 and 20 μg of HEK293 stable cell membranes, respectively.

### cAMP Assay

Peptide ligands were assayed for cAMP inhibition in the HEK293-stable mouse KOR cell line in a 384-well format and triplicates according to the Cisbio protocol. Briefly, 5 μL containing 2000 cells per well were incubated with 5 μL of peptide solutions prepared using 2× in 1× stimulation buffer and forskolin (10 μM final). Following the incubation of peptide ligands at 37 °C for 30 min, cryptate-labeled cAMP and cAMP d2-labeled antibody (5 μL of each) were added to the mixture and further incubated for at least 1 h at room temperature. Subsequently, cAMP levels were quantified on a Flexstation 3 (Molecular Devices, USA) using homogeneous time-resolved fluorescence resonance energy transfer (HTRF) and the ratio 665/620 nm. Antagonism of CSD-CH_2(1,8)_-NH_2_ at KOR was evaluated by the Schild regression analysis as previously described.^50^ Briefly, the concentration–response curves of U50,488 were measured in the presence and absence of CSD-CH_2(1,8)_-NH_2_. The cells were pretreated with CSD-CH_2(1,8)_-NH_2_ (0.3, 1, 3, and 10 μM) for 30 min at 37 °C, followed by co-incubation with U50,488 for an additional 30 min at 37 °C. The reaction was terminated by the addition of 5 μL of cAMP-d2 antibody and 5 μL of cAMP Eu cryptate. After 1 h of incubation at 37 °C, fluorescence was measured on a Flexstation 3 and quantified using the 665/620 nm ratio. The samples were measured in technical triplicates.

### BRET Assay

The BRET assay was performed as previously described.^50^ Briefly, human β-arrestin-2-nano luciferase (Nluc) and mouse KOR-EGFP were transiently expressed in HEK293 cells following co-transfection in a 1:10 ratio. 16 h post-transfection, 50,000 cells in 100 μL of phenol red-free DMEM with 10% FBS per well were transferred into white clear bottom 96-well plates and incubated overnight. On the day of assay, cells were incubated for 1 h at 37 °C in phenol red- and serum-free DMEM. Furimazine (Promega, USA) and peptide ligands were diluted 4× and 1:50 in Hank’s balanced salt solution (HBSS), respectively. Prior to establishing the baseline and measuring the kinetic BRET, furimazine was incubated for 5 min at 37 °C. The ability of peptide ligands to induce β-arrestin recruitment was assessed by measuring light emissions for EGFP (510 nm) and Nluc (460 nm) over 35 min using a Flexstation 3. The BRET ratio was calculated as emission EGFP (510 nm)/emission Nluc (460 nm). The ligand-induced BRET was calculated by subtracting the BRET ratio of the ligand from the BRET ratio of the HBSS. Relative BRET quantification was determined by calculating the mean value of ligand-induced BRET for each peptide ligand from 311 to 2411 s (35 min), which was then divided by the mean value of ligand-induced BRET of dynA_1–13_-NH_2_ set as 100.

### Animals and Drug Administration

Breeding, euthanasia, and all procedures of animal handling were performed according to the regulations of animal care and welfare. Experiments were carried out in accordance with the European Communities Council Directive of 24 November 1986 (86/609/EEC). Behavioral experiments were performed in male CD1 mice (8–10 weeks old) purchased from Janvier Labs (Le Genest-Saint-Isle, France). All animal care and experimental procedures were in accordance with the ethical guidelines for the animal welfare standards of the European Communities Council Directive (2010/63/EU) and were approved by the Committee of Animal Care of the Austrian Federal Ministry of Science and Research. Mice were group-housed in a temperature-controlled specific pathogen-free room with a 12 h light/dark cycle and with free access to food and water. U50,488, nor-BNI, and CSD-CH_2(1,8)_-NH_2_ were prepared in sterile physiological 0.9% saline solution. Test compounds or vehicle (saline) were s.c. administered in a volume of 10 μL/g body weight.

### Isolation of DRG Neurons

Wild-type C57BL/6 mice (13–17 weeks of age) were euthanized by cervical dislocation preceded by anesthesia by exposure to isoflurane. DRGs from all spinal levels were excised and transferred to DMEM containing 1% streptomycin/penicillin and 1% l-glutamine treated with 1 mg/mL collagenase (Sigma, Austria) and 3 mg/mL dispase II (Roche, Austria) for 60 min at 37 °C. Digested DRGs were then mechanically dissociated with a Pasteur pipette, centrifuged at 1000 rpm for 5 min, and plated onto 12 mm glass coverslips previously coated with 100 mg/mL poly-d-lysine (Sigma). DRG neurons were cultured in DMEM supplemented with 100 mg/mL streptomycin/penicillin, 1% l-glutamine, and 100 ng/mL mouse nerve growth factor (Alomone Labs, Israel). Neurons were cultured at 37 °C and 5% CO_2_ for 15–30 h.

### Single-Cell Calcium Microfluorimetry

The coverslips were incubated with Fura-2 AM ester (Biotium, USA) for 30 min at 37 °C and 5% CO_2_ before placement in glass-bottom 35 mm dishes in an extracellular solution (in mM: 145 NaCl, 5 KCl, 10 glucose, 10 HEPES, 1.25 CaCl_2_, 1 MgCl_2_, pH 7.4, and 300 mOsm). After a recovery period of 10 min, dishes were mounted onto an Olympus IX73-inverted microscope (Olympus, Tokyo, Japan) and imaged using a 10× objective. Cells were continuously superfused with the extracellular solution using a software-controlled eight-channel, gravity-driven, common-outlet system (ALA Scientific Instruments Inc, USA). This superfusion was switched to different solutions after 2 min of baseline recording. A positive control detecting viable cells was added at the end of each recording using depolarization by an extracellular solution with 60 mM KCl (isotonic replacement of NaCl). Fura-2 was alternatingly excited for 30 ms by a 340 nm LED (50 mW, used at 100%) and by a 385 nm LED (1435 mW, used at 5%) using an Omicron LEDHub (Laserage-Laserprodukte GmbH, Germany). Fluorescence emission was long-pass-filtered at 495 nm, and pairs of images were acquired at a rate of 1 Hz with a 4.2-megapixel 16 bit CCD camera (6.5 μm pixel edge length, 18.8 mm sensor diameter, Prime BSI; Teledyne Photometrics, USA). The hardware was controlled by the μManager 1.4 plugin in ImageJ.^51^ The background intensity was subtracted before calculating the ratio between the fluorescence emitted when the dye was excited at 340 nm and 385 nm (F340/F385 nm). The time course of this ratio was analyzed for regions of interest adapted to individual cells.

### Tail-Flick Assay

The radiant heat tail-flick test was performed using an UB 37360 Ugo Basile analgesiometer (Ugo Basile s.r.l., Varese, Italy) as described previously.^52^ The reaction time required by the mouse to remove its tail after application of the radiant heat was measured and defined as the tail-flick latency (in seconds). Tail-flick latencies were measured before and after s.c. administration of saline (control) or U50,488 (5 mg/kg) (i.e., 30 and 60 min) (test latency, TL). In antagonist studies, CSD-CH_2(1,8)_-NH_2_ (20 mg/kg) or nor-BNI (10 mg/kg) were s.c. administered 15 min or 24 h, respectively, before U50,488 (5 mg/kg). A cutoff time of 10 s was used in order to minimize tissue damage. Each experimental group included five to six mice. The antinociceptive effect (as the percentage of the maximum possible effect, % MPE) was calculated according to the formula = [(TL – BL)/(cutoff time – BL)] × 100 where TL represents the test latency and BL is the basal latency.

### Rotarod Test

The rotarod test was performed using the accelerating rotarod treadmill (Acceler Rota-Rod 7650, Ugo Basile s.r.l., Varese, Italy) for mice (diameter 3.5 cm) as described previously.^53^ Animals were habituated to the equipment in two training sessions (30 min apart) 1 day before testing. On the experimental day, mice were placed on the rotarod, and treadmill was accelerated from 4 to 40 rpm over a period of 5 min. The time spent on the drum was recorded for each mouse before (baseline) and at 30 min after s.c. administration of saline (control) or U50,488 (5 mg/kg). In antagonist studies, CSD-CH_2(1,8)_-NH_2_ (20 mg/kg) or nor-BNI (10 mg/kg) were s.c. administered 15 min or 24 h, respectively, before U50,488 (5 mg/kg). Decreased latencies to fall in the rotarod test indicate impaired motor performance. A 300 s cutoff time was used. Each experimental group included five to six mice. The rotarod data are expressed as percentage (%) changes from the rotarod latencies obtained before (baseline, BL) and after drug administration (test, TL) calculated as 100 × (TL/BL).

### Data and Statistical Analysis

Data analysis was performed with GraphPad Prism (GraphPad Software, USA). The potency (EC_50_) and maximum efficacy (*E*_max_) values of peptide ligands were derived from functional data fitted to three-parameter non-linear regression curves constrained to a bottom of zero and a hill slope of 1. Graphs were normalized to 100%, i.e., the highest concentration (10 μM) of dynA_1–13_-NH_2_. For the Schild plot analysis, data were normalized to 100%, i.e., the highest concentration (10 μM) of U50,488 and fitted to three-parameter non-linear regression curves constrained to a bottom of zero. The logarithm of the concentration–ratio (*A*′/*A* – 1) was plotted against the logarithm of the respective concentration of antagonist CSD-CH_2(1,8)_-NH_2_ (B) to derive the pA2 value (functional inhibitory affinity).

Data from radioligand binding studies were fitted using the three-parameter one-site competition binding equation with constrained *K*_d_ and concentration of [^3^H]-diprenorphine used in the assay (1 nM) to obtain IC_50_ values. Calculated IC_50_ values were then used to obtain inhibition constants (*K*_i_) using the Cheng and Prusoff equation. Data were normalized to specific binding of [^3^H]-diprenorphine without competing ligands and are reported as maximum percentage (100%), which denotes an average of 7–8 pmol/mg for KOR, MOR, and DOR. In vivo data were statistically evaluated using one-way ANOVA with Tukey’s multiple comparisons post hoc test with significance set at *P* < 0.05.

## Supporting Information Available

Pharmacological data of CSD ligands from functional BRET and radioligand binding assays, HPLC data of the synthesized CSD ligands, MALDI-TOF MS data of CSD ligands, and ^1^H NMR data of all CSD ligands (PDF)
Molecular formula string spreadsheet for all synthetic compounds (CSV)

## Abbreviations

BRET: bioluminescence resonance energy transfer
cAMP: cyclic adenosine monophosphate
CSD: cysteine-stapled dynorphin
DOR: δ-opioid receptor
DRG: dorsal root ganglion
dynA: dynorphin A
EGFP: enhanced green fluorescence protein
ESI-MS: electrospray ionization mass spectrometry
GPCR: G protein-coupled receptor
KOR: κ-opioid receptor
MALDI-MS: matrix-assisted laser desorption ionization mass spectrometry
MOR: μ-opioid receptor
nor-BNI: norbinaltorphimine
RP-HPLC: reversed-phase high-performance liquid chromatography
s.c.: subcutaneous
