# Supplementary material for: Development of a Selective Peptide κ-Opioid Receptor Antagonist by Late-Stage Functionalization with Cysteine Staples
Source: J Med Chem. 2023 Aug 26;66(17):11843–54. doi: 10.1021/acs.jmedchem.3c00426 (PMC10510397; doi:10.1021/acs.jmedchem.3c00426)
Supplement: Supplementary file 1 — jm3c00426_si_001.pdf [file jm3c00426_si_001.pdf]

# Supporting Information

## Development of a Selective Peptide κ-opioid Receptor Antagonist by Late-Stage Functionalization with Cysteine Staples

Edin Muratspahić▲, Andrew M. White▲, Cosmin I. Ciotu, Nadine Hochrainer, Nataša Tomašević, Johannes Koehbach, Richard J. Lewis, Mariana Spetea, Michael J. M. Fischer, David J. Craik*, Christian W. Gruber*

***Correspondence to** Christian W. Gruber, Email: christian.w.gruber@meduniwien.ac.at or David J. Craik, Email: d.craik@imb.uq.edu.au

▲these authors contributed equally

**This PDF file includes:**

Table S1

Figures S1 to S23

**Table S1. Characterization and purity analysis of CSD peptide ligands determined by MALDI-TOF mass spectrometry and RP-HPLC.**

| Peptide | $[M+H]^+$ (calculated | $[M+H]^+$ (observed) | RP-HPLC Purity (%) |
|---|---|---|---|
| CSD-ox$_{(1,8)}$-OH | 1679.9 | 1679.9 | 99.3 |
| CSD-ox$_{(1,10)}$-OH | 1695.9 | 1696.0 | 99.8 |
| CSD-CH$_{2(1,8)}$-OH | 1693.9 | 1693.9 | 97.8 |
| CSD-CH$_{2(1,10)}$-OH | 1709.9 | 1710.0 | 97.0 |
| CSD-mXYL$_{(1,8)}$-OH | 1784.0 | 1784.0 | 98.1 |
| CSD-mXYL$_{(1,10)}$-OH | 1800.0 | 1800.0 | 98.4 |
| CSD-tet$_{(1,8)}$-OH | 1759.9 | 1760.1 | 98.2 |
| CSD-tet$_{(1,10)}$-OH | 1775.9 | 1776.0 | 97.5 |
| CSD-ace$_{(1,8)}$-OH | 1735.9 | 1736.0 | 98.5 |
| CSD-ace$_{(1,10)}$-OH | 1752.0 | 1752.0 | 97.5 |
| CSD-CH$_{2(1,8)}$-NH$_2$ | 1692.9 | 1693.0 | 97.3 |
| CSD-CH$_{2(1,10)}$-NH$_2$ | 1709.0 | 1709.0 | 97.0 |
| CSD-ace$_{(1,8)}$-NH$_2$ | 1734.9 | 1735.0 | 96.3 |

Monoisotopic masses of peptides obtained by ESI- and/or MALDI-MS are shown. RP-HPLC purity (%) was determined by integration (area under the curve) of the analytical traces at 214 nm.

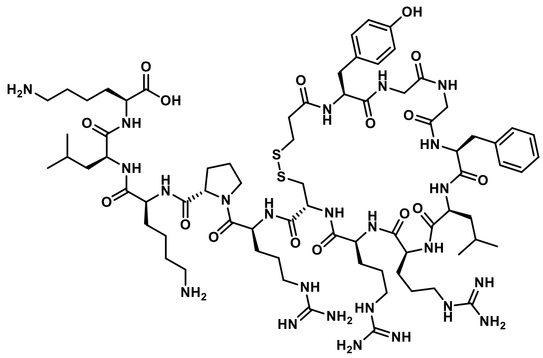

CSD-ox$_{(1-8)}$-OH

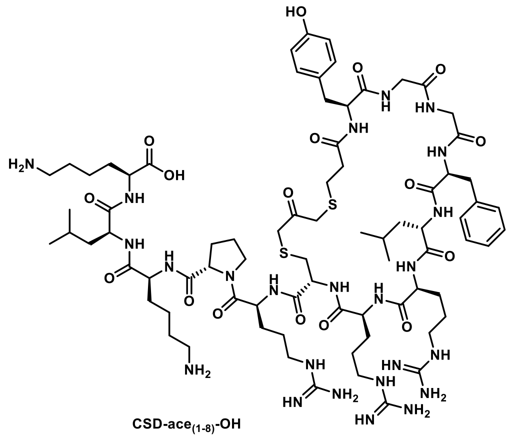

CSD-ace$_{(1-8)}$-OH

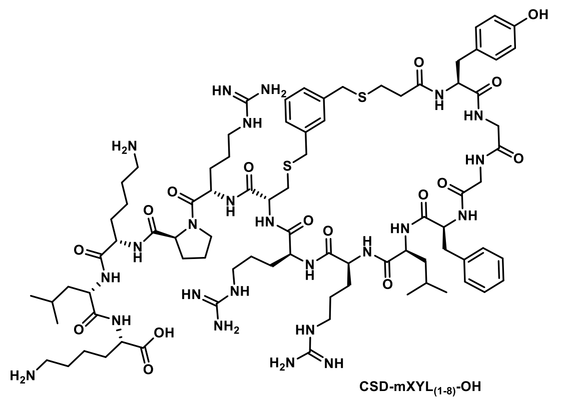

CSD-mXYL$_{(1-8)}$-OH

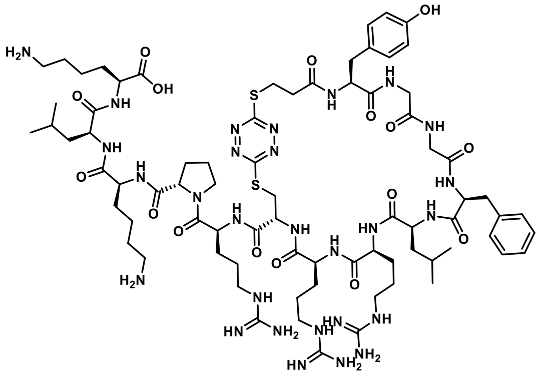

CSD-tet$_{(1-8)}$-OH

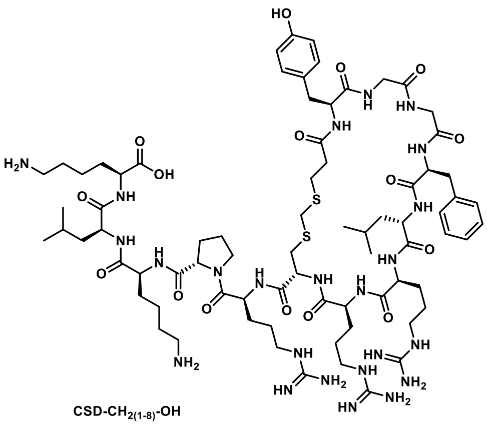

CSD-CH$_{2(1-8)}$-OH

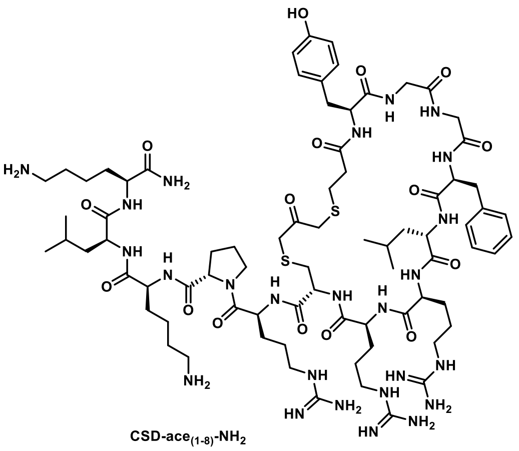

CSD-ace$_{(1-8)}$-NH$_2$

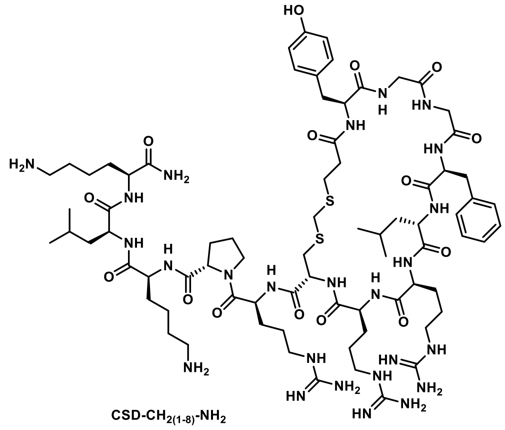

CSD-CH$_{2(1-8)}$-NH$_2$

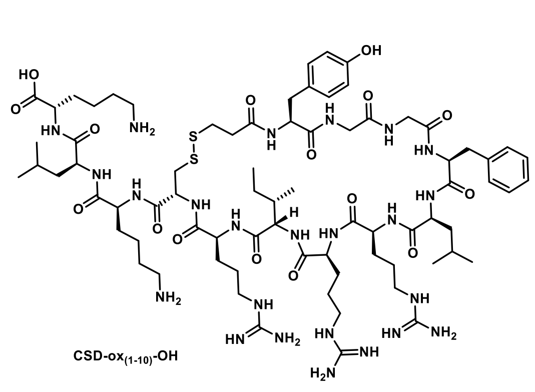

CSD-ox$_{(1-10)}$-OH

**Figure S1. Structures of synthesized dyn A$_{1\text{-}13}$ cysteine staples comprising COOH or NH$_2$ at the C-terminus**

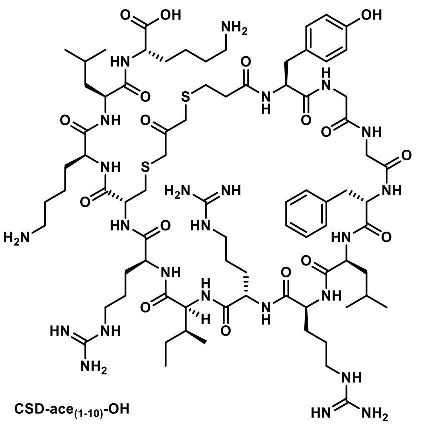

CSD-ace$_{(1\text{-}10)}$-OH

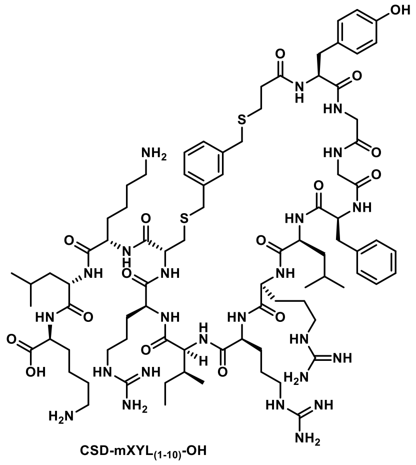

CSD-mXYL$_{(1\text{-}10)}$-OH

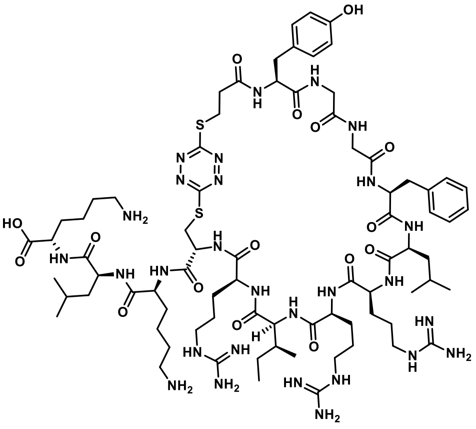

CSD-tet$_{(1\text{-}10)}$-OH

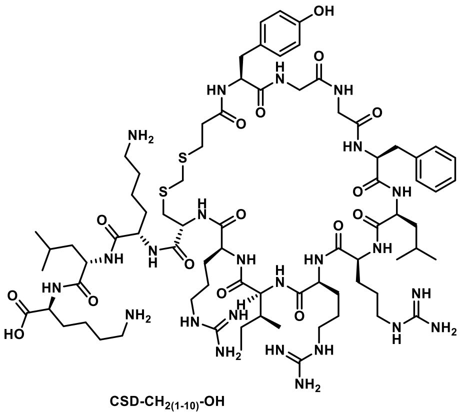

CSD-CH$_{2(1\text{-}10)}$-OH

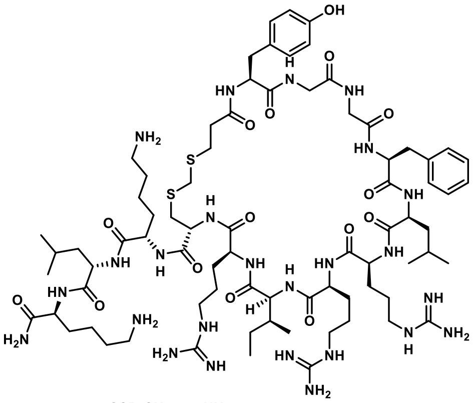

CSD-CH$_{2(1\text{-}10)}$-NH$_2$

**Figure S1 (continued). Structures of synthesized dyn $A_{1\text{-}13}$ cysteine staples comprising COOH or $NH_2$ at the C-terminus**

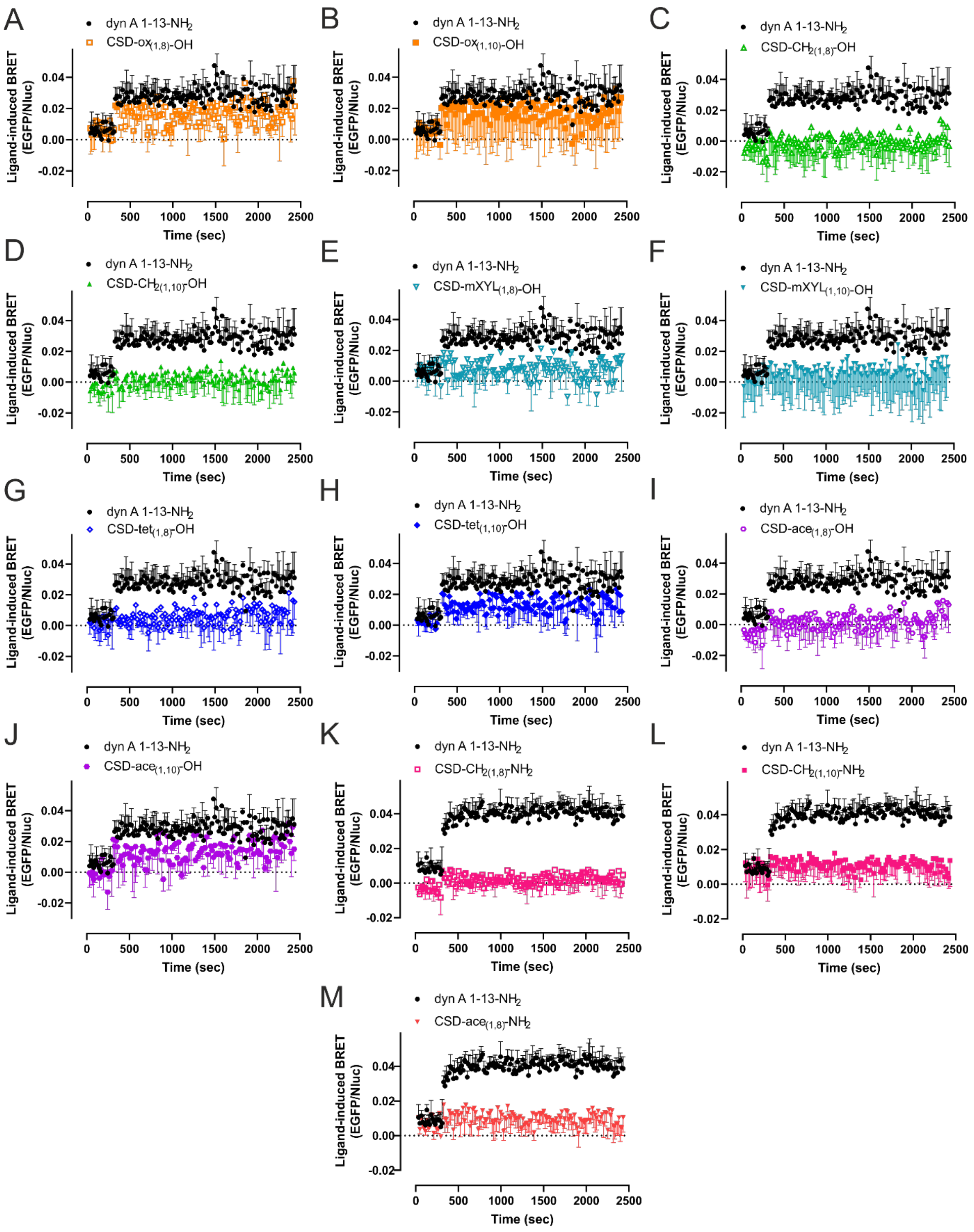


**Figure S2. Kinetic bioluminescence resonance energy transfer (BRET) reported by nanoLuc.** Substances at a concentration of 10 µM were applied after 300 s to measure β-arrestin recruitment at the KOR of (**A**) CSD-ox$_{(1,8)}$-OH (n=3, orange empty squares), (**B**) CSD-ox$_{(1,10)}$-OH (n=3, orange squares), (**C**) CSD-CH$_{2(1,8)}$-OH (n=3, green empty triangles), (**D**) CSD-CH$_{2(1,10)}$-OH (n=3, green triangles), (**E**) CSD-mXYL$_{(1,8)}$-OH (n=3, cyan empty inverted triangles), (**F**) CSD-mXYL$_{(1,10)}$-OH (n=3, cyan inverted triangles), (**G**) CSD-tet$_{(1,8)}$-OH (n=3, blue empty diamonds), (**H**) CSD-tet$_{(1,10)}$-OH (n=3, blue diamonds), (**I**) CSD-ace$_{(1,8)}$-OH (n=3, violet empty hexagons) and (**J**) CSD-ace$_{(1,10)}$-OH (n=3, violet hexagons), CSD-CH$_{2(1,8)}$-NH$_2$ (n=3, pink empty squares), CSD-CH$_{2(1,10)}$-NH$_2$ (n=3, pink squares), CSD-ace$_{(1,8)}$-NH$_2$ (n=3, red inverted triangles). Dynorphin (dyn) A$_{1\text{-}13}$-NH$_2$ served as control (n=3, black circles in all panels).

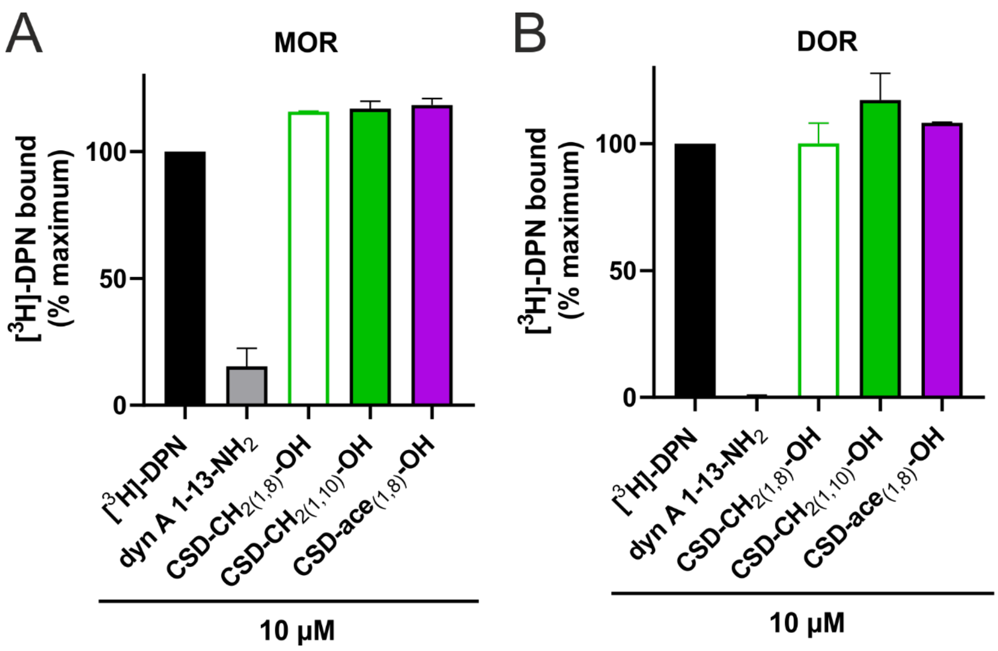


**Figure S3. Subtype selectivity of CSD-OH analogs.** CSD-OH peptide ligands were measured for displacing 1 nM of tritiated diprenorphine ([$^3$H]-DPN, black bar) in radioligand binding studies in HEK293 cells stably expressing (**A**) mouse µ-opioid receptor (MOR) and (**B**) mouse δ-opioid receptor (DOR) (n=2). Dynorphin (dyn) $A_{1\text{-}13}$-$NH_2$ (black bars) served as positive control (10 µM, n=2).

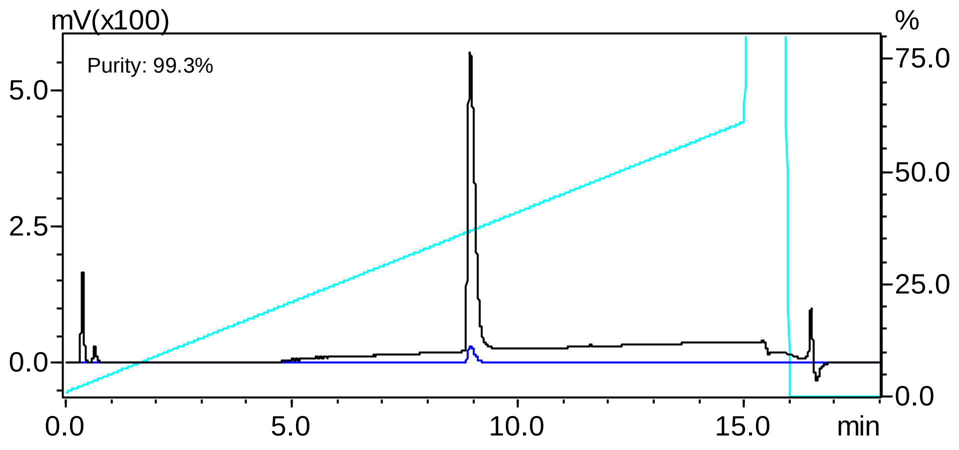


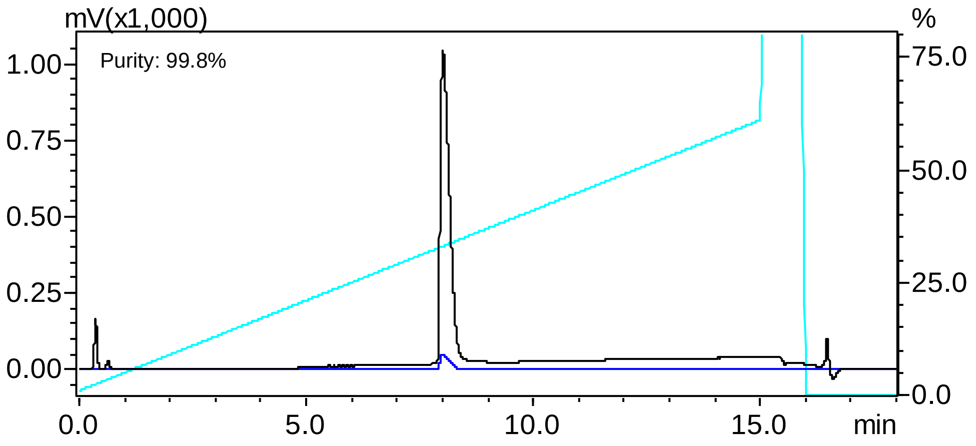


**Figure S4. HPLC of CSD-ox$_{(1,8)}$-OH (top) and CSD-ox$_{(1,10)}$-OH (bottom).** UV at 215 nm (black chromatogram) and 280 (blue chromatogram). Linear gradient (cyan) with 0-60% solvent B over 15 min and a flow rate of 0.6 mL/min on a $C_{18}$ column (1.6 µm, 100 Å, 50 × 2.1 mm) was used.

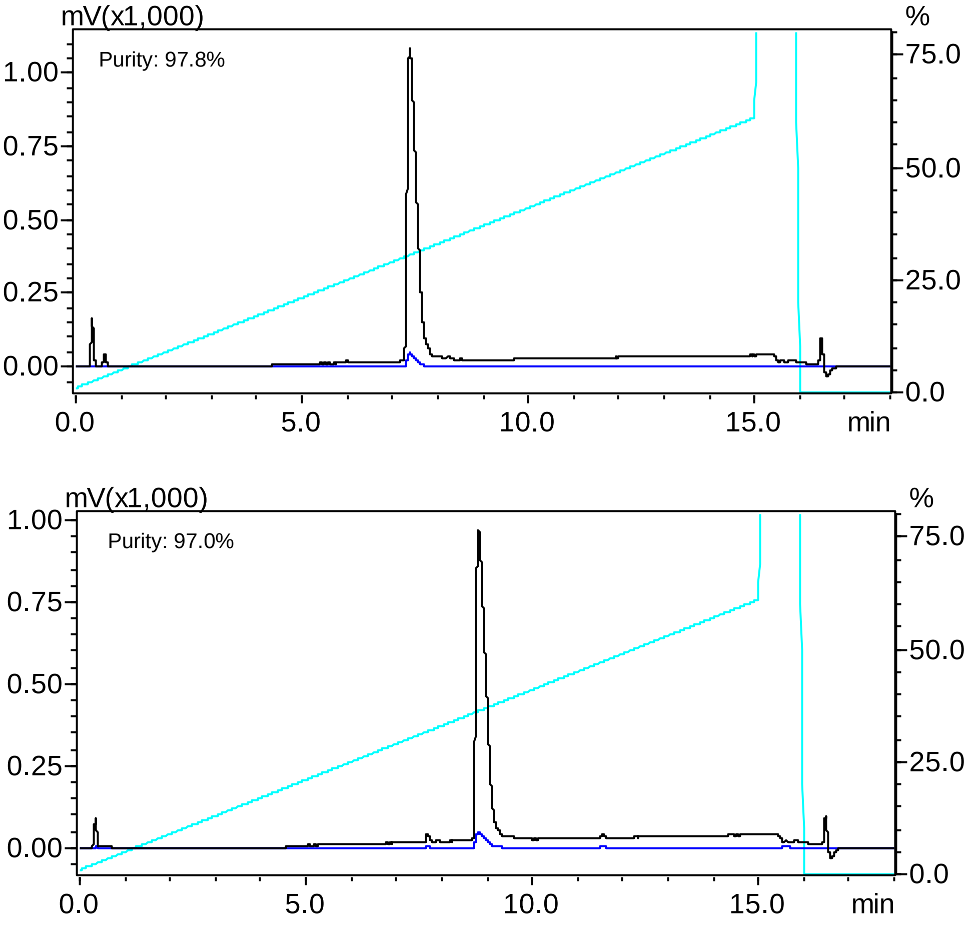


**Figure S5. HPLC of CSD-$CH_{2(1,8)}$-OH (top) and CSD-$CH_{2(1,10)}$-OH (bottom).** UV at 215 nm (black chromatogram) and 280 (blue chromatogram). Linear gradient (cyan) with 0-60% solvent B over 15 min and a flow rate of 0.6 mL/min on a $C_{18}$ column (1.6 µm, 100 Å, 50 × 2.1 mm) was used.

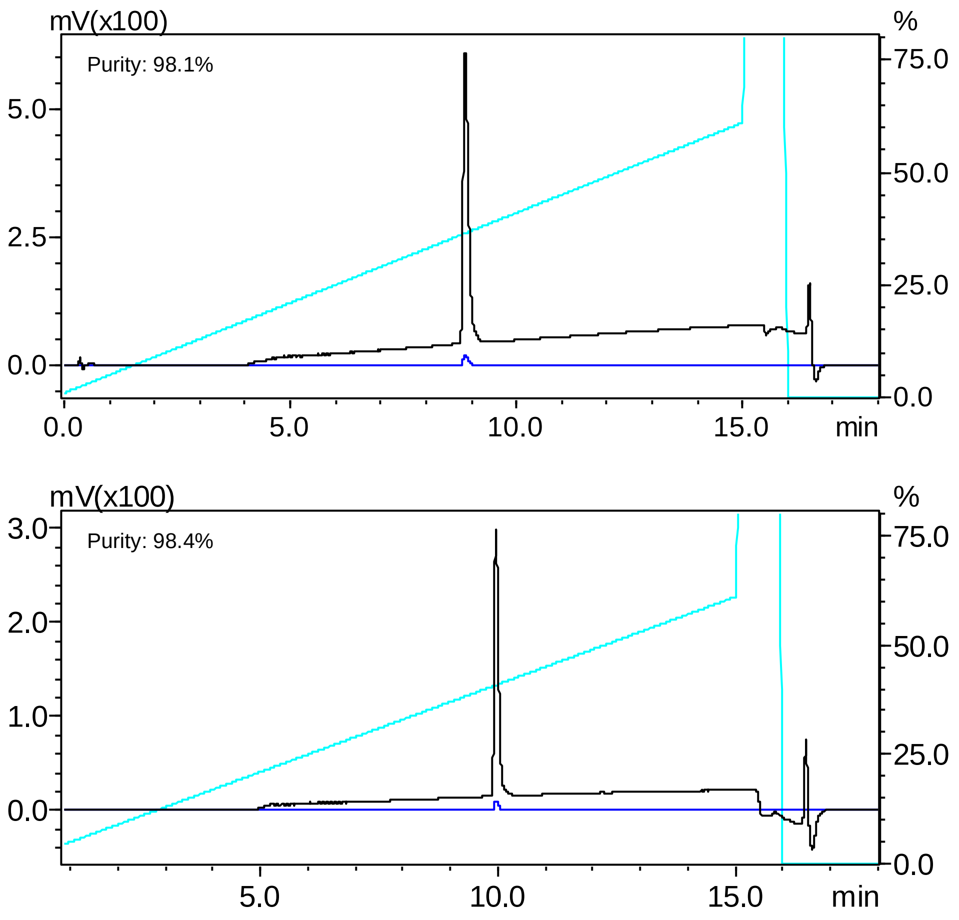


**Figure S6. HPLC of CSD-mXYL$_{(1,8)}$-OH (top) and CSD-mXYL$_{(1,10)}$-OH (bottom).** UV at 215 nm (black chromatogram) and 280 (blue chromatogram). Linear gradient (cyan) with 0-60% solvent B over 15 min and a flow rate of 0.6 mL/min on a $C_{18}$ column (1.6 µm, 100 Å, 50 × 2.1 mm) was used.

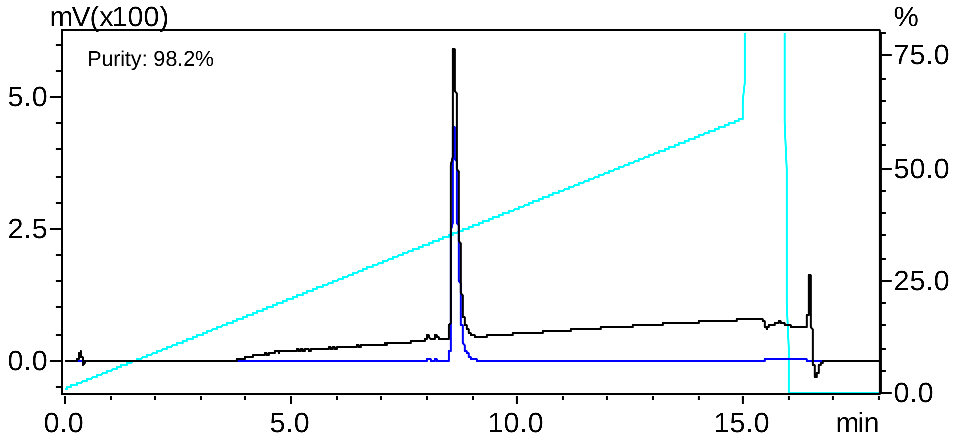


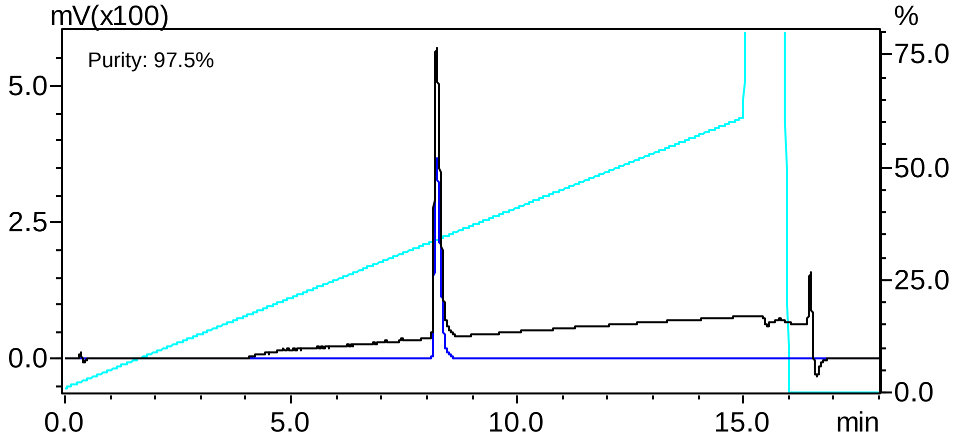


**Figure S7. HPLC of CSD-tet$_{(1,8)}$-OH (top) and CSD-tet$_{(1,10)}$-OH (bottom).** UV at 215 nm (black chromatogram) and 280 (blue chromatogram). Linear gradient (cyan) with 0-60% solvent B over 15 min and a flow rate of 0.6 mL/min on a $C_{18}$ column (1.6 µm, 100 Å, 50 × 2.1 mm) was used.

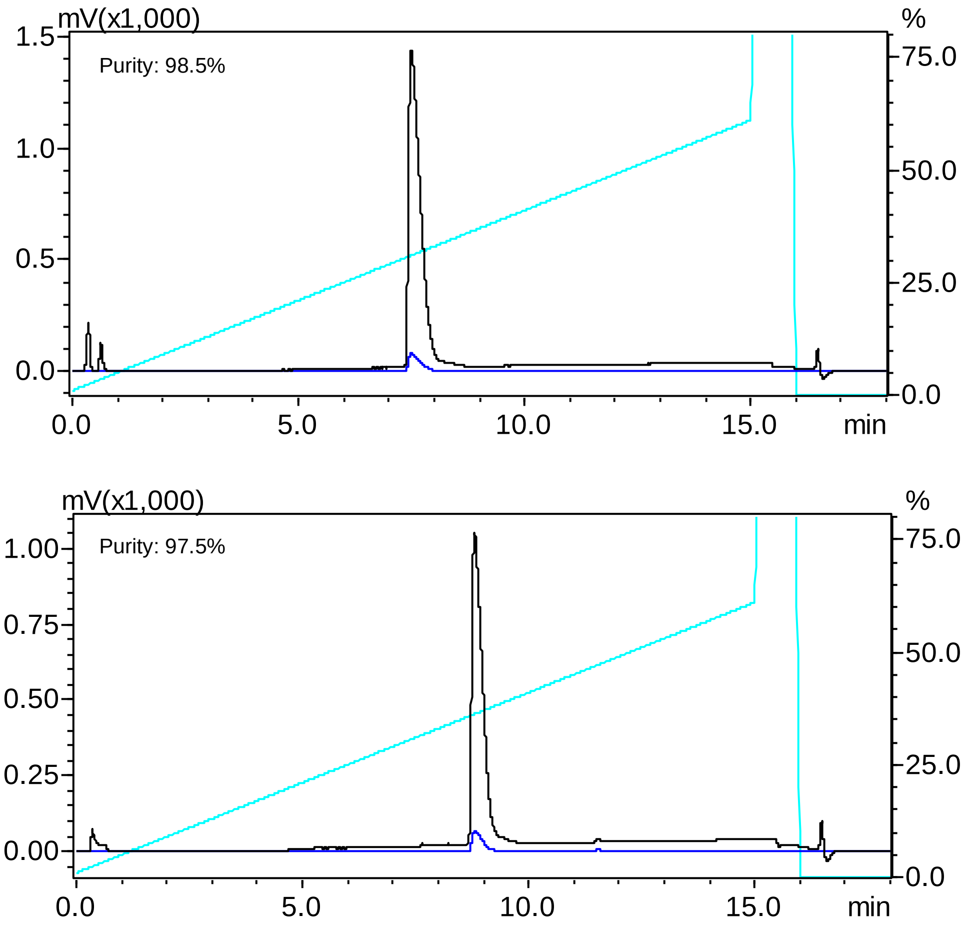


**Figure S8. HPLC of CSD-ace$_{(1,8)}$-OH (top) and CSD-ace$_{(1,10)}$-OH (bottom).** UV at 215 nm (black chromatogram) and 280 (blue chromatogram). Linear gradient (cyan) with 0-60% solvent B over 15 min and a flow rate of 0.6 mL/min on a $C_{18}$ column (1.6 µm, 100 Å, 50 × 2.1 mm) was used.

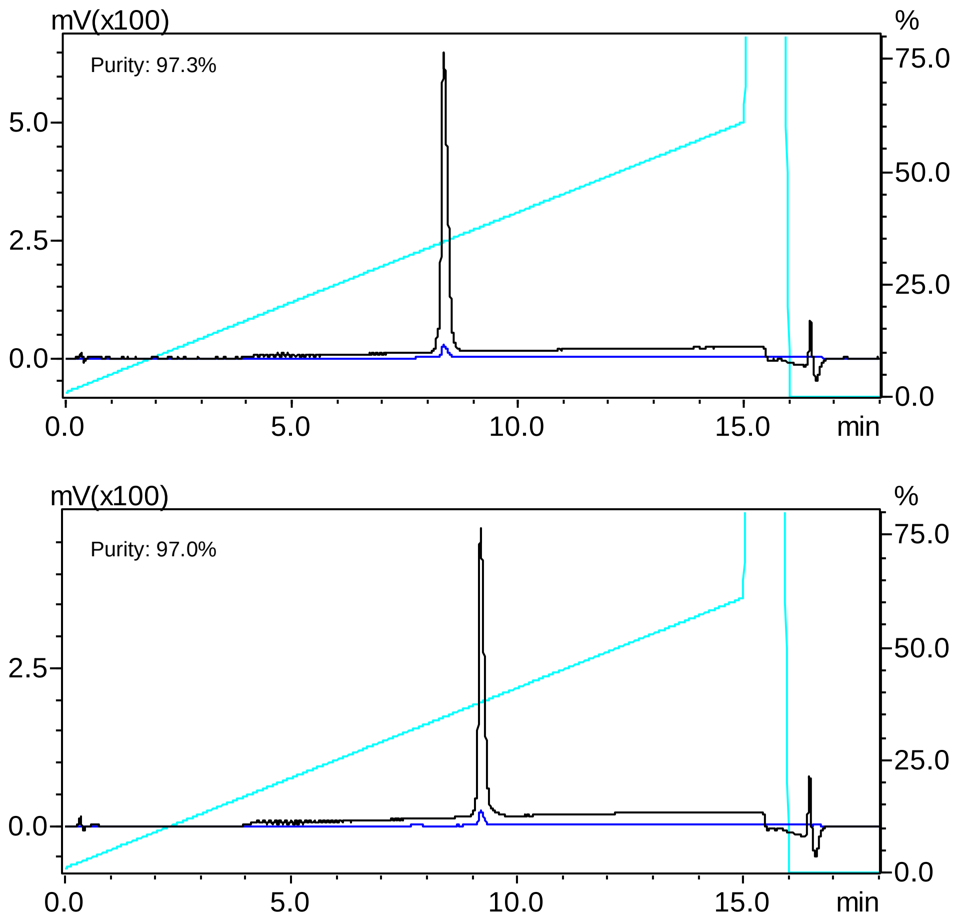


**Figure S9. HPLC of CSD-$CH_{2(1,8)}$-$NH_2$ (top) and CSD-$CH_{2(1,10)}$-$NH_2$ (bottom).** UV at 215 nm (black chromatogram) and 280 (blue chromatogram). Linear gradient (cyan) with 0-60% solvent B over 15 min and a flow rate of 0.6 mL/min on a $C_{18}$ column (1.6 µm, 100 Å, 50 × 2.1 mm) was used.

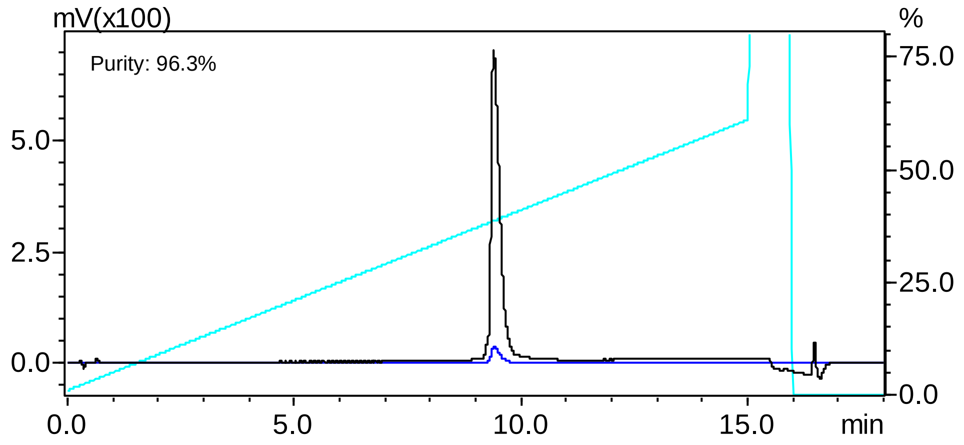


**Figure S10.** HPLC of CSD-ace$_{(1,8)}$-NH$_2$. UV at 215 nm (black chromatogram) and 280 (blue chromatogram). Linear gradient (cyan) with 0-60% solvent B over 15 min and a flow rate of 0.6 mL/min on a C$_{18}$ column (1.6 µm, 100 Å, 50 × 2.1 mm) was used.

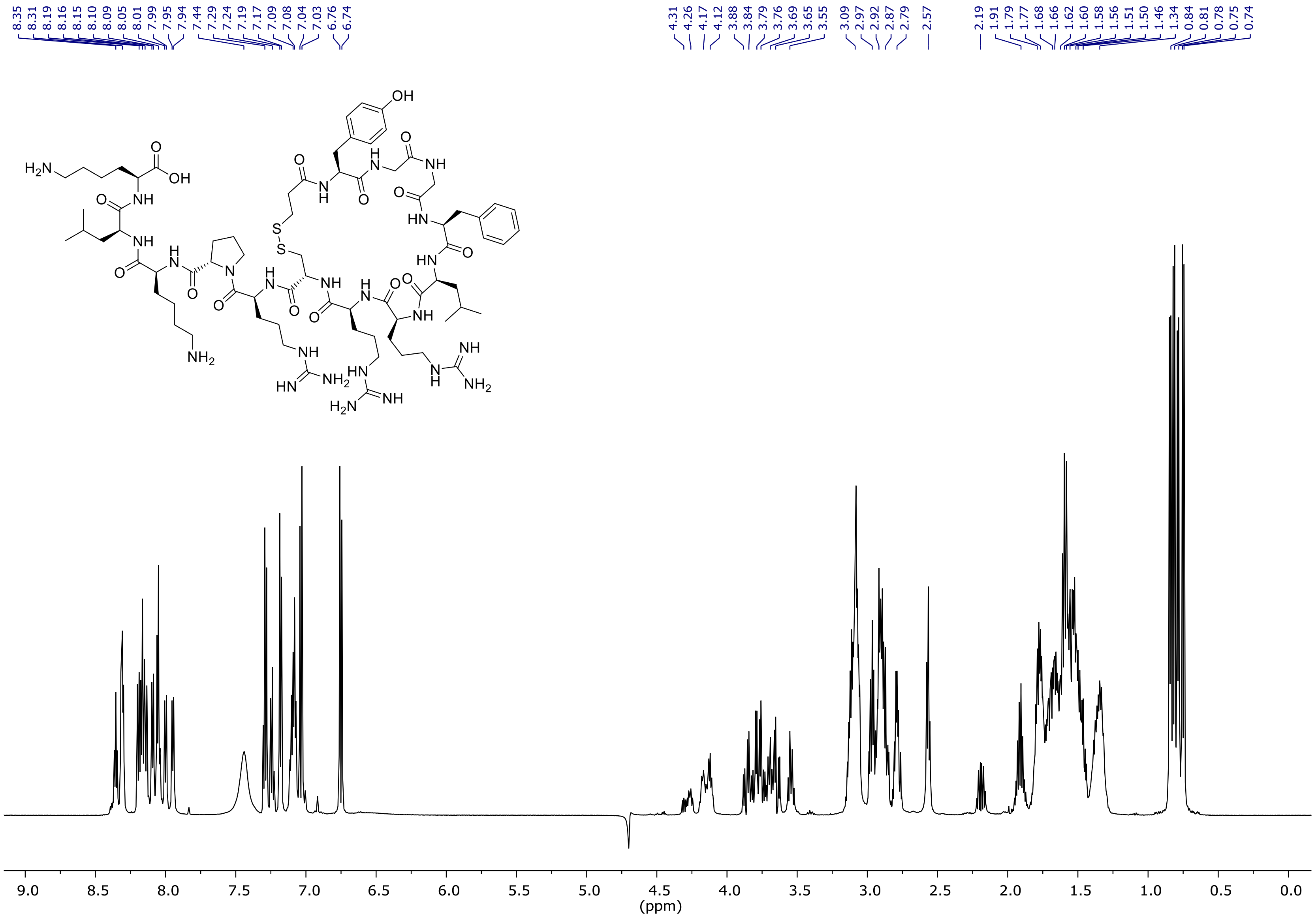

**Figure S11.** $^{1}$**H NMR spectrum of CSD-ox**$_{(1,8)}$**-OH at 600 MHz in** $H_2O/D_2O$ **(9:1, v/v)**

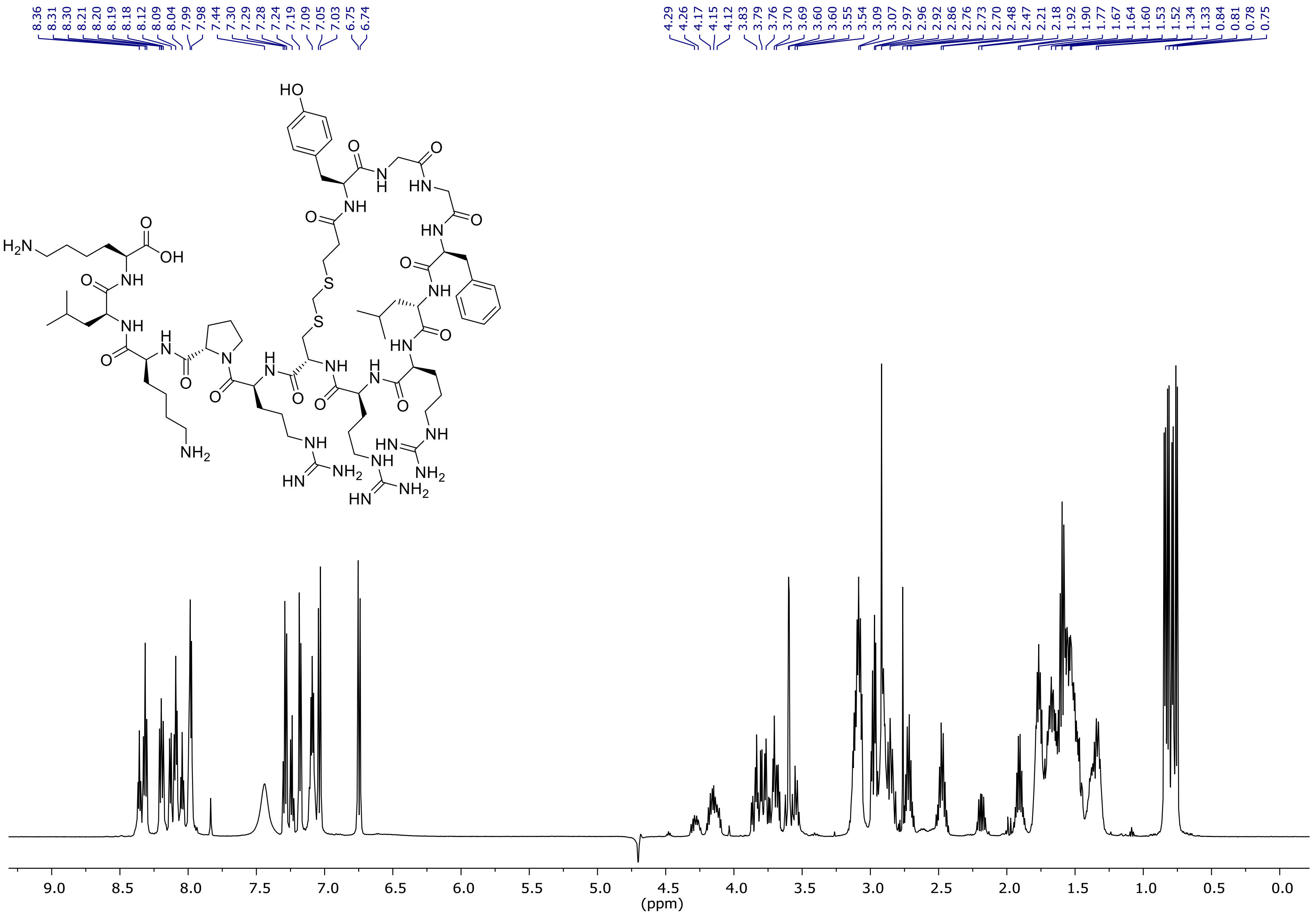


**Figure S12.** **$^{1}$H NMR spectrum of CSD-CH$_{2(1,8)}$-OH at 600 MHz in $H_2O/D_2O$ (9:1, v/v)**

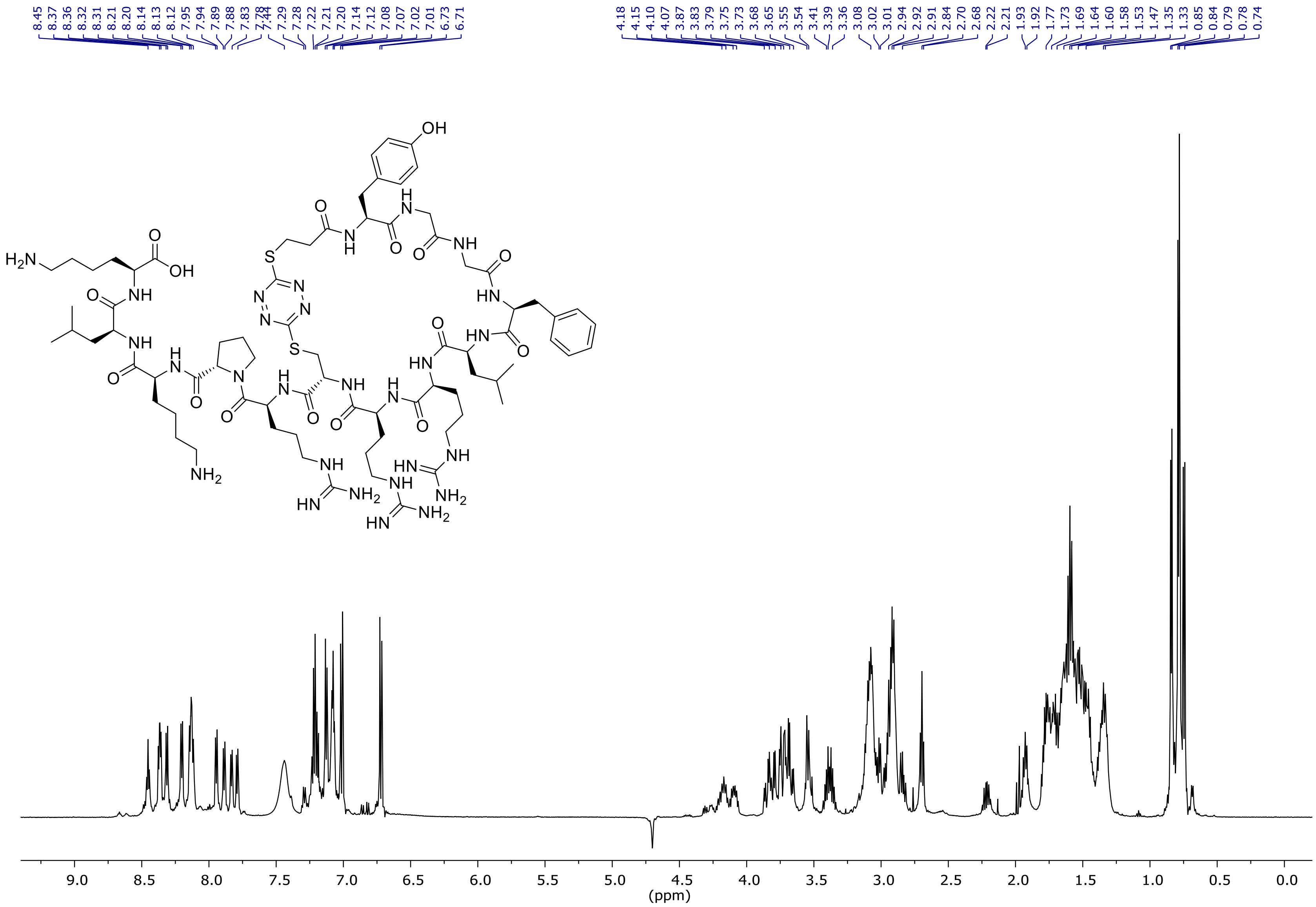


**Figure S13.** $^{1}H$ **NMR spectrum of CSD-tet$_{(1,8)}$-OH at 600 MHz in $H_2O/D_2O$ (9:1, v/v)**

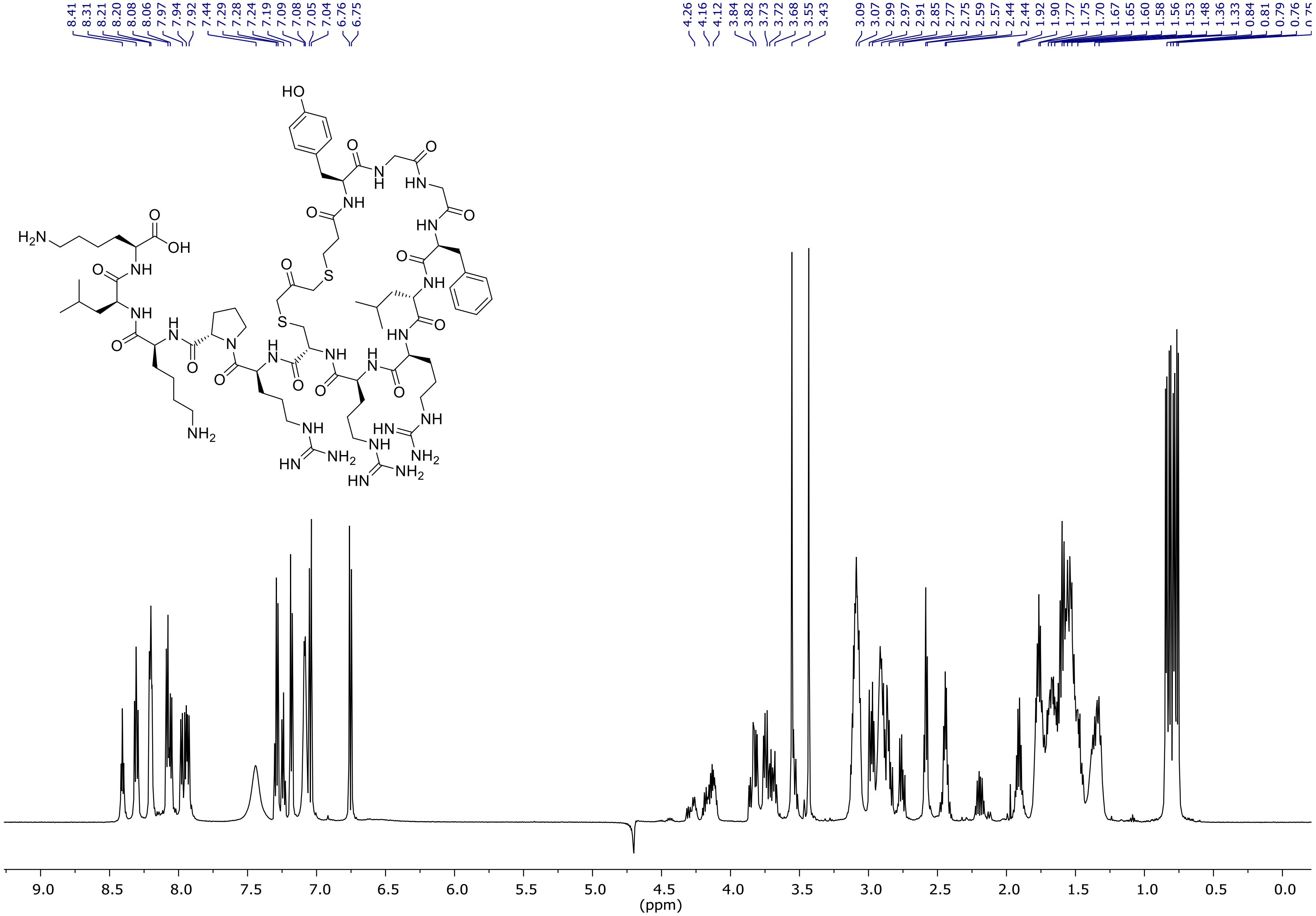

**Figure S14. $^{1}$H NMR spectrum of CSD-ace(1,8)-OH at 600 MHz in $H_2O/D_2O$ (9:1, v/v)**

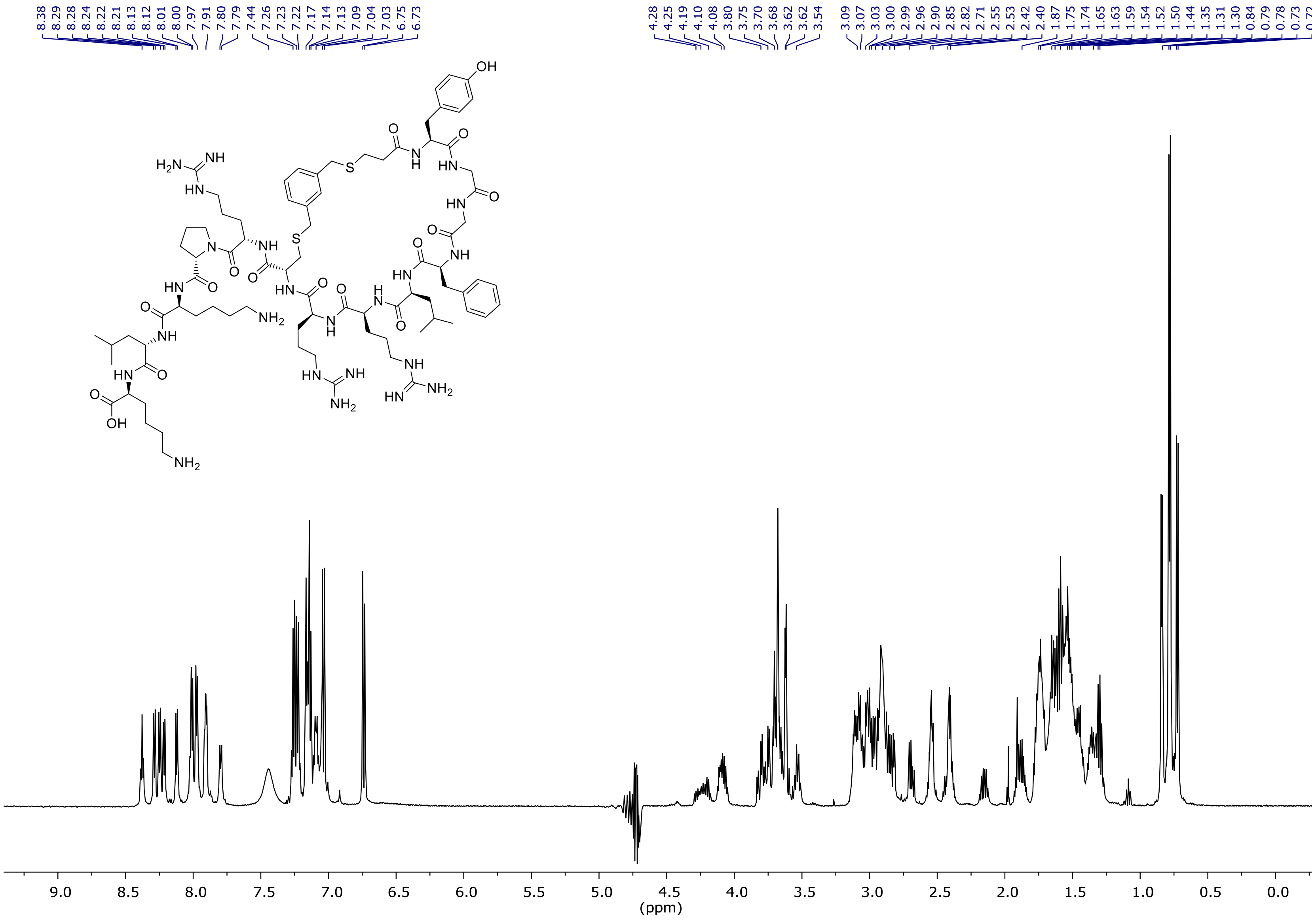


**Figure S15.** $^{1}$H NMR spectrum of CSD-mXYL$_{(1,8)}$-OH at 600 MHz in $H_2O/D_2O$ (9:1, v/v)

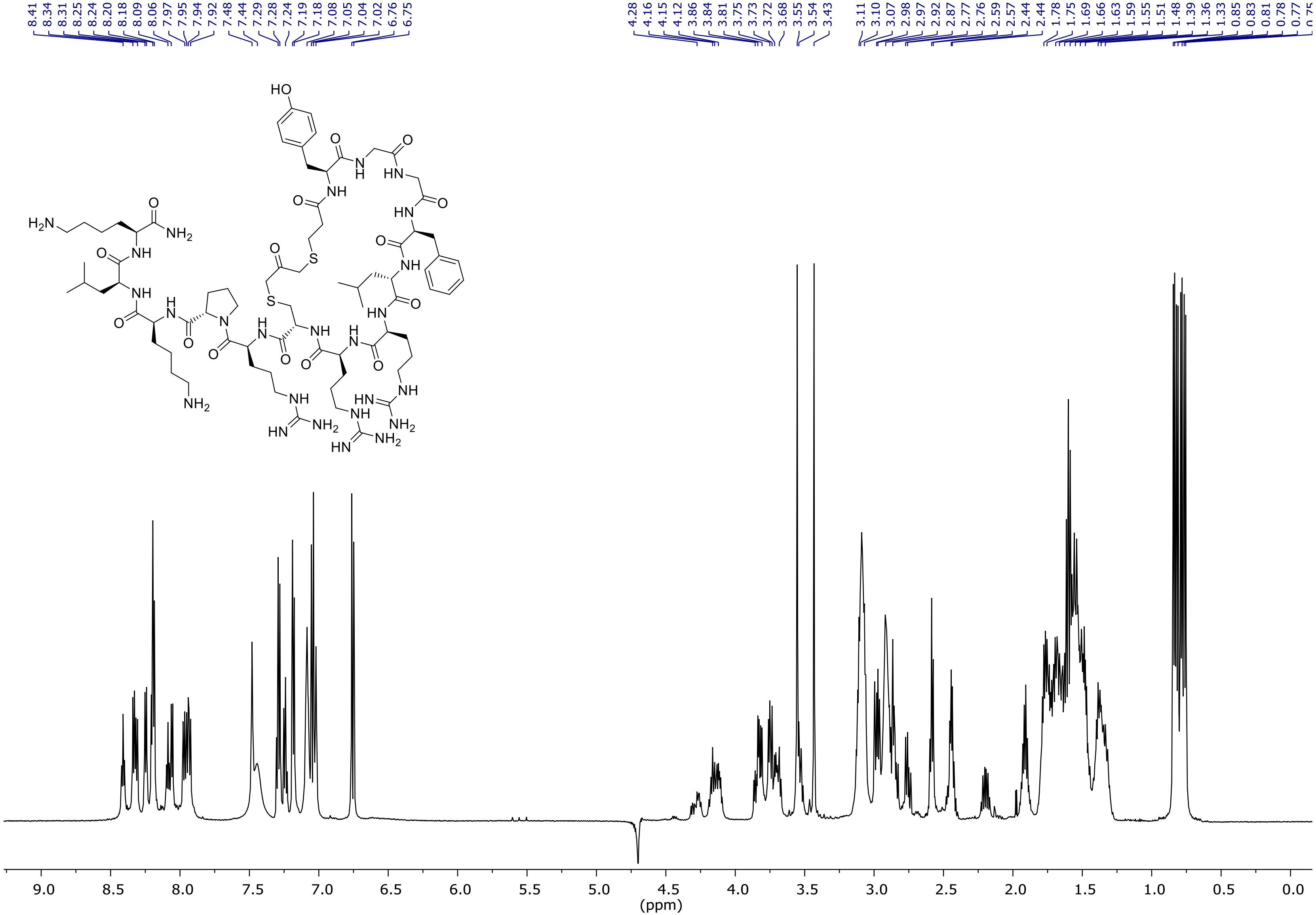


**Figure S16.** **$^{1}$H NMR spectrum of CSD-ace$_{(1,8)}$-NH$_2$ at 600 MHz in $H_2O/D_2O$ (9:1, v/v)**

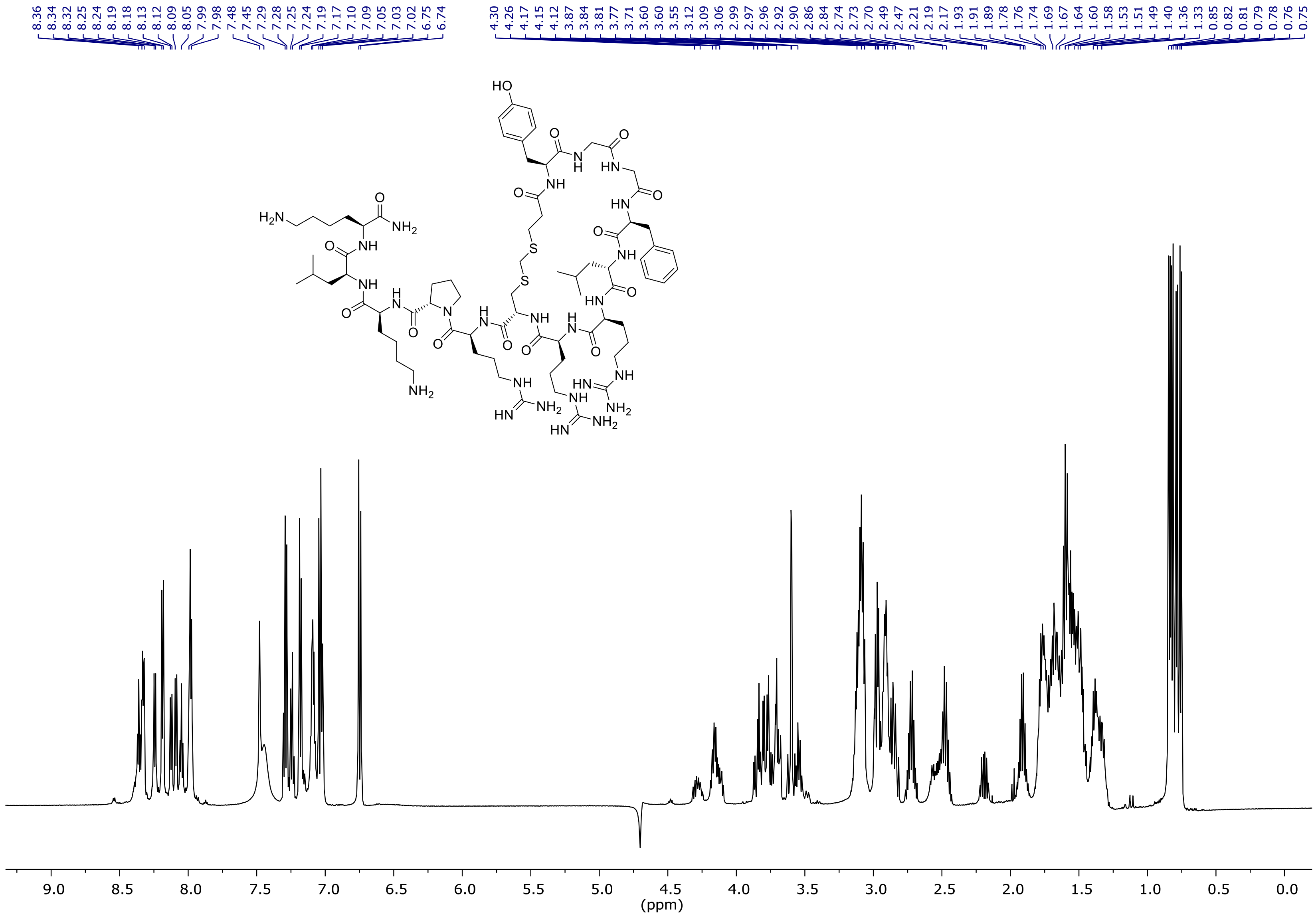


**Figure S17.** $^{1}H$ **NMR spectrum of CSD-CH$_{2(1,8)}$-NH$_2$ at 600 MHz in $H_2O/D_2O$ (9:1, v/v)**

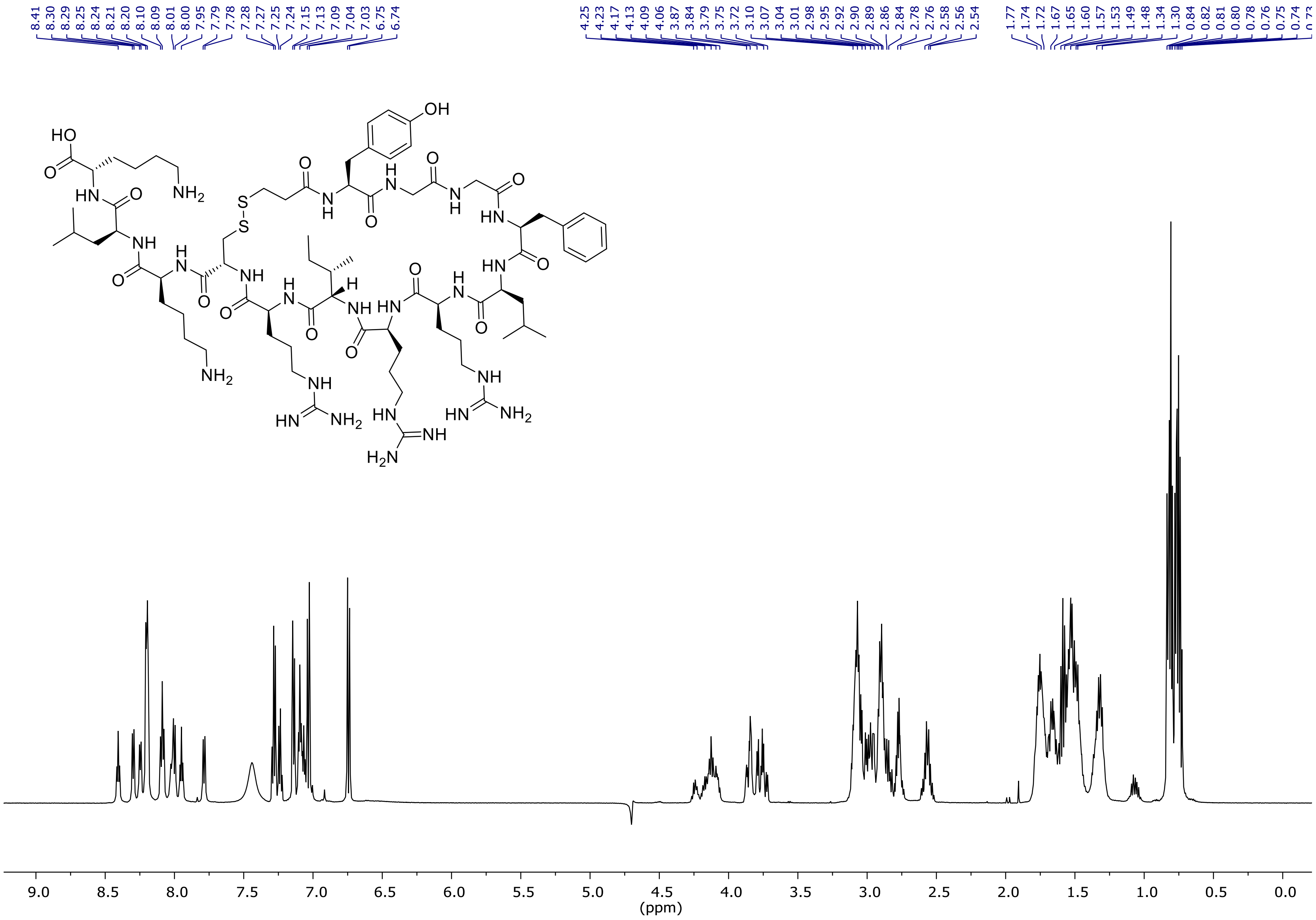


**Figure S18.** **$^{1}$H NMR spectrum of CSD-ox(1,10)-OH at 600 MHz in $H_2O/D_2O$ (9:1, v/v)**

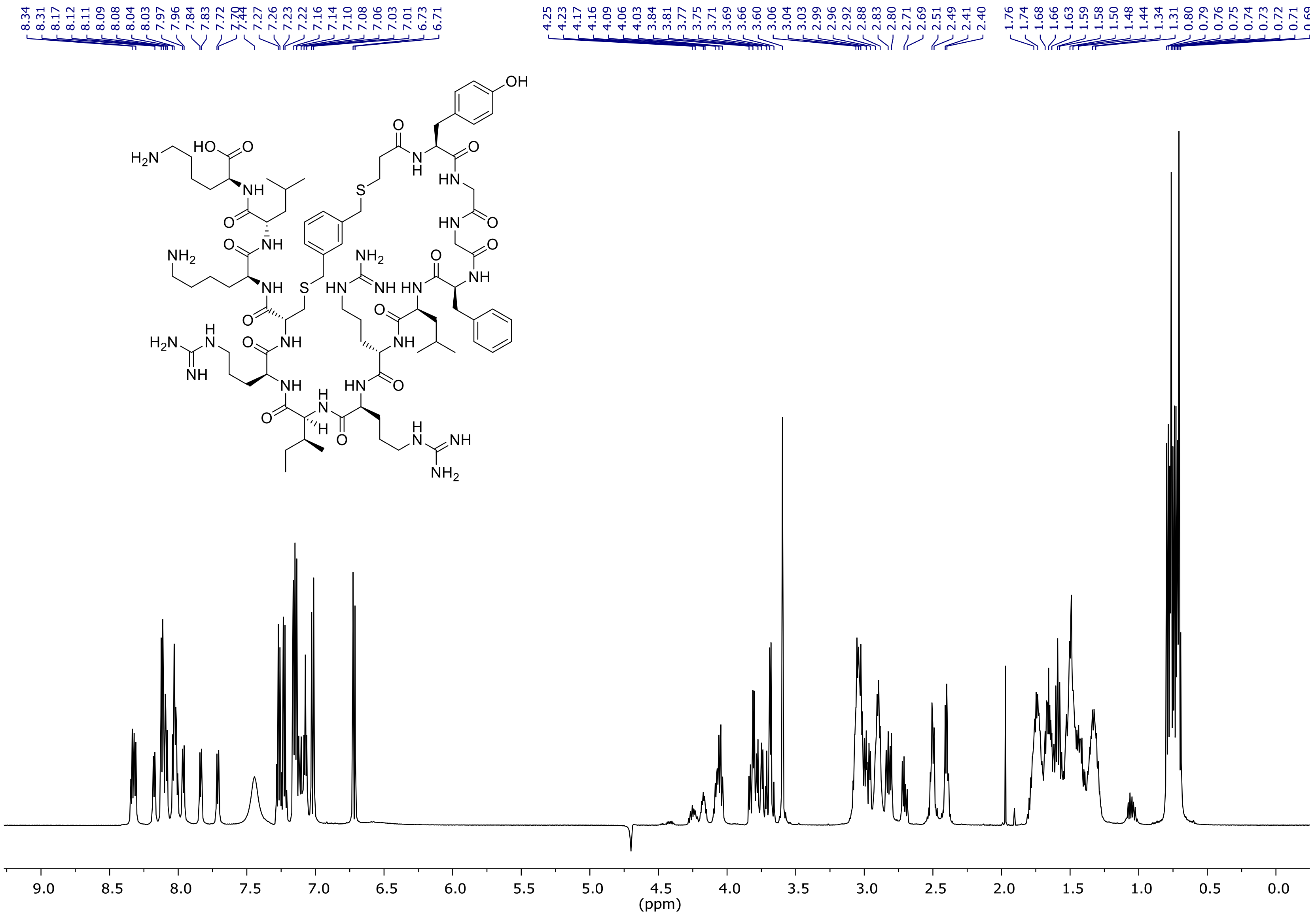

**Figure S19.** **¹H NMR spectrum of CSD-mXYL$_{(1,10)}$-OH at 600 MHz in $H_2O/D_2O$ (9:1, v/v)**

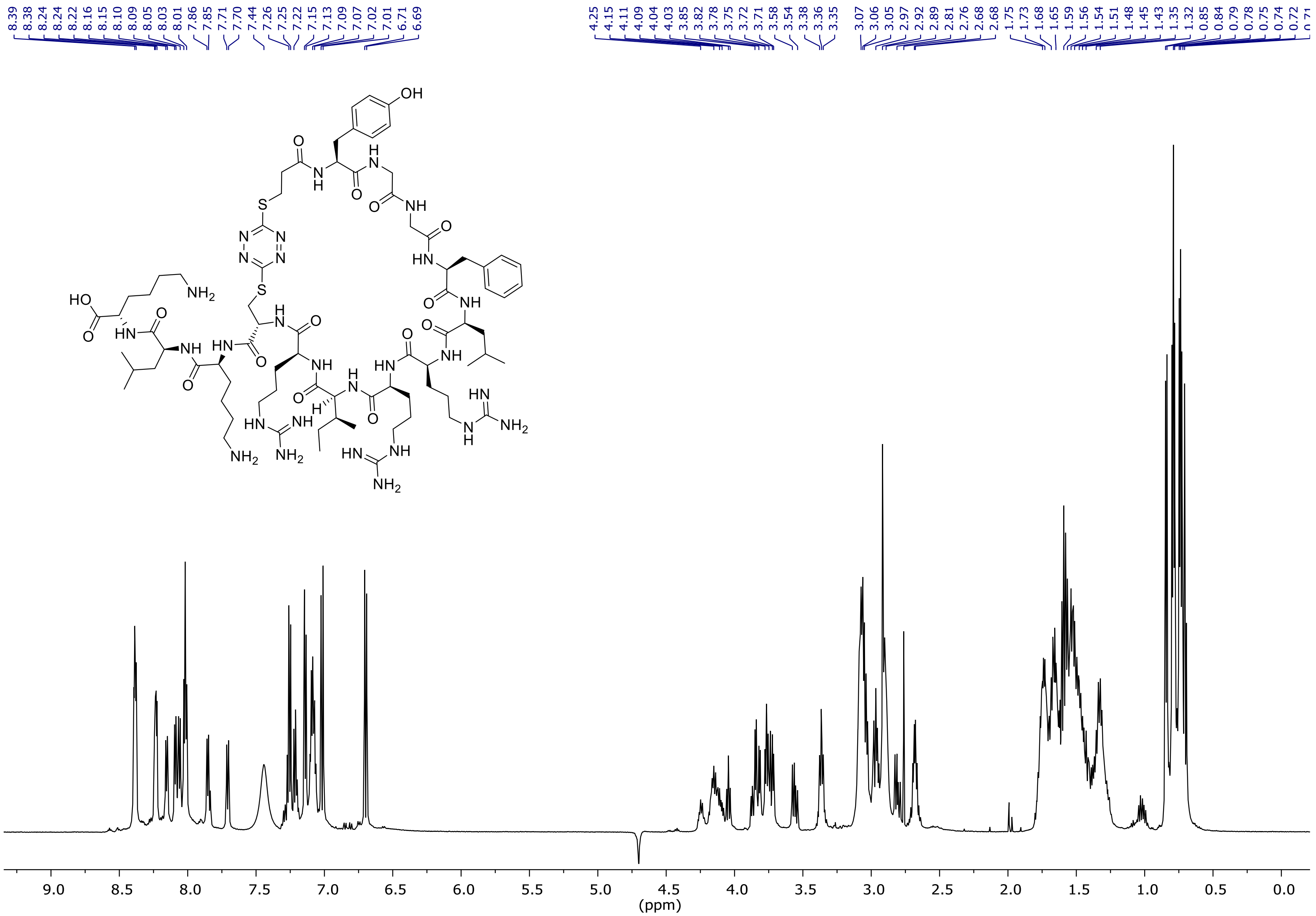

**Figure S20. $^{1}$H NMR spectrum of CSD-tet$_{(1,10)}$-OH at 600 MHz in $H_2O/D_2O$ (9:1, v/v)**

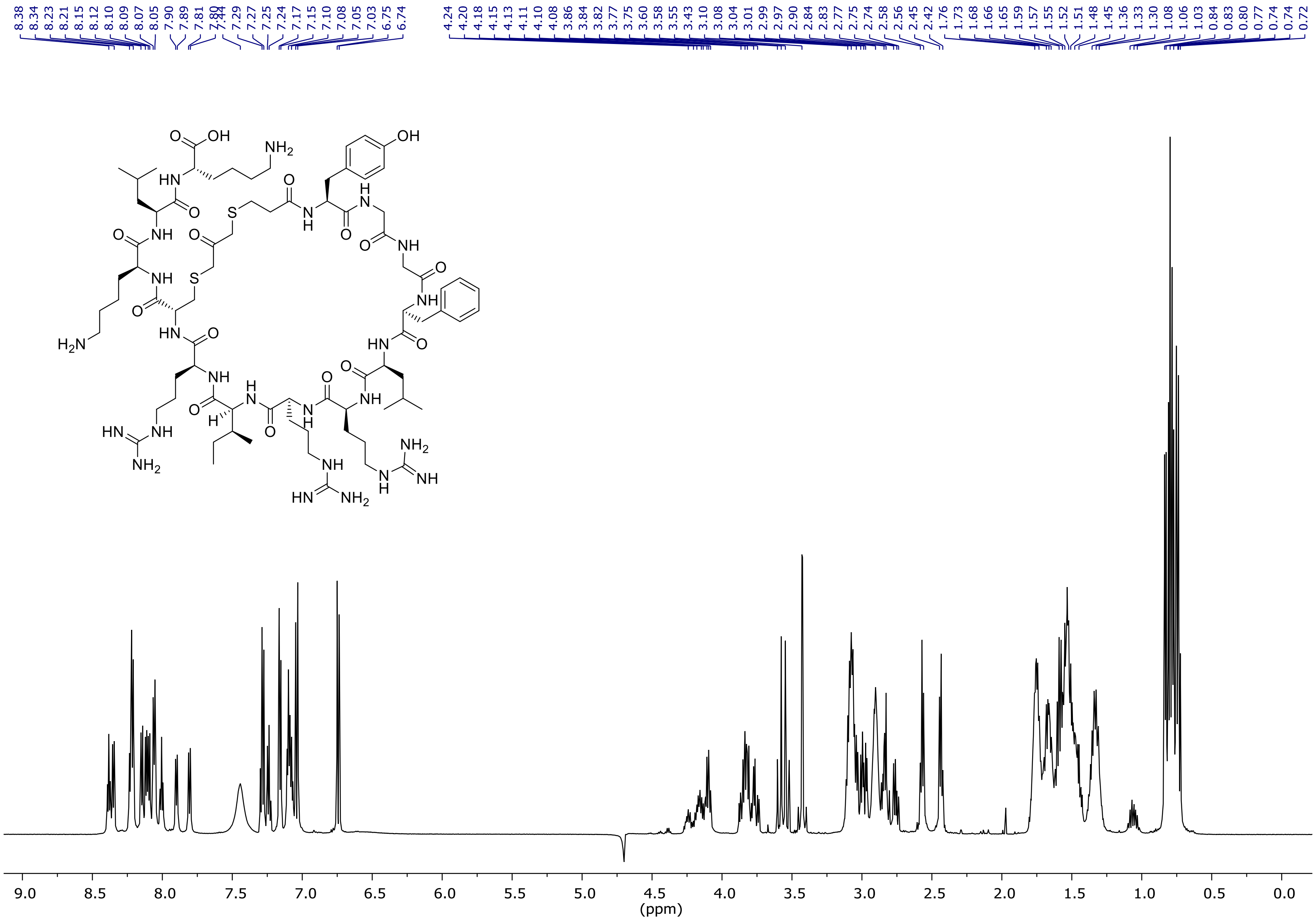


**Figure S21.** **$^{1}$H NMR spectrum of CSD-ace$_{(1,10)}$-OH at 600 MHz in $H_2O/D_2O$ (9:1, v/v)**

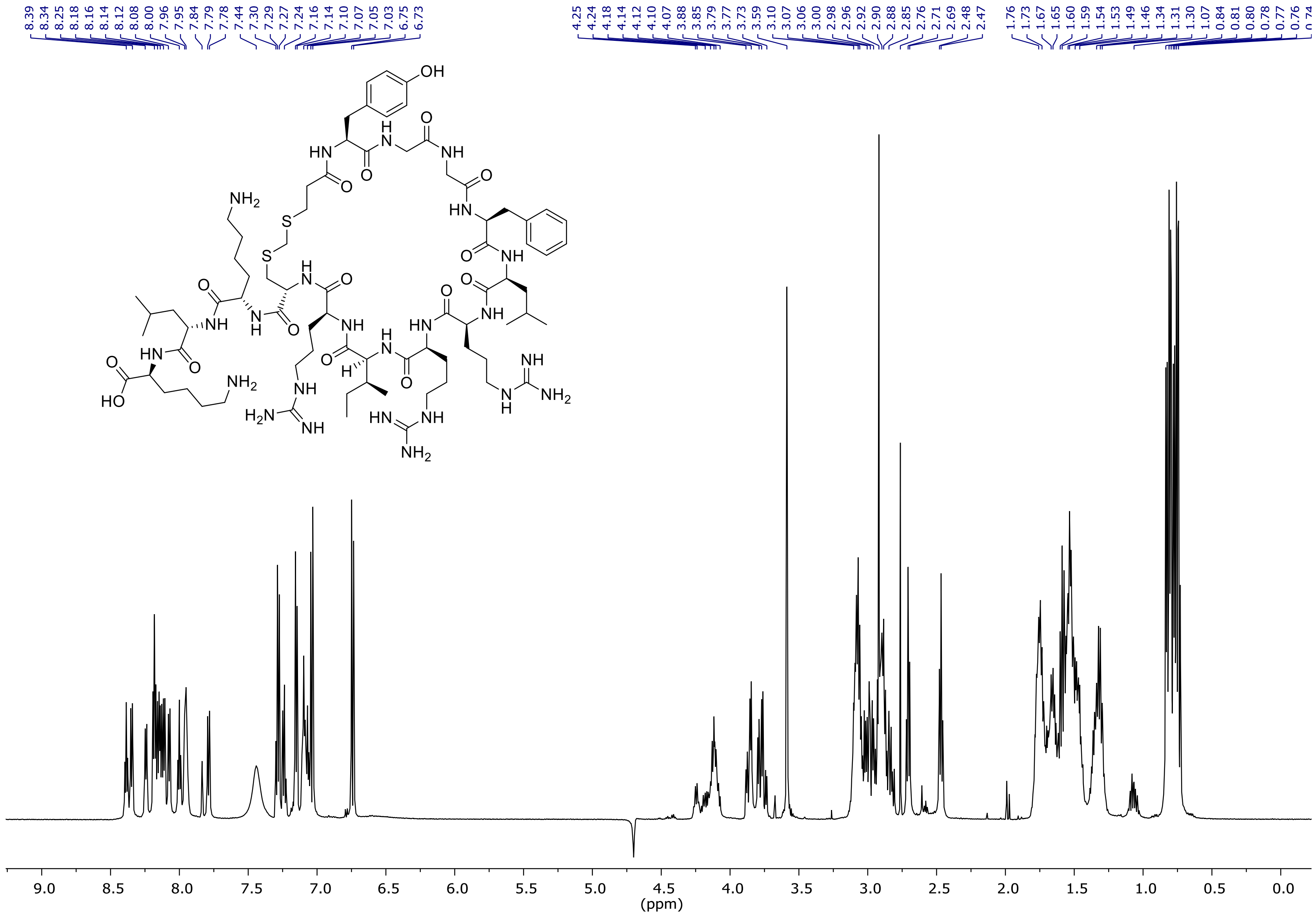


**Figure S22.** $^{1}$**H NMR spectrum of CSD-CH$_{2(1,10)}$-OH at 600 MHz in H$_2$O/D$_2$O (9:1, v/v)**

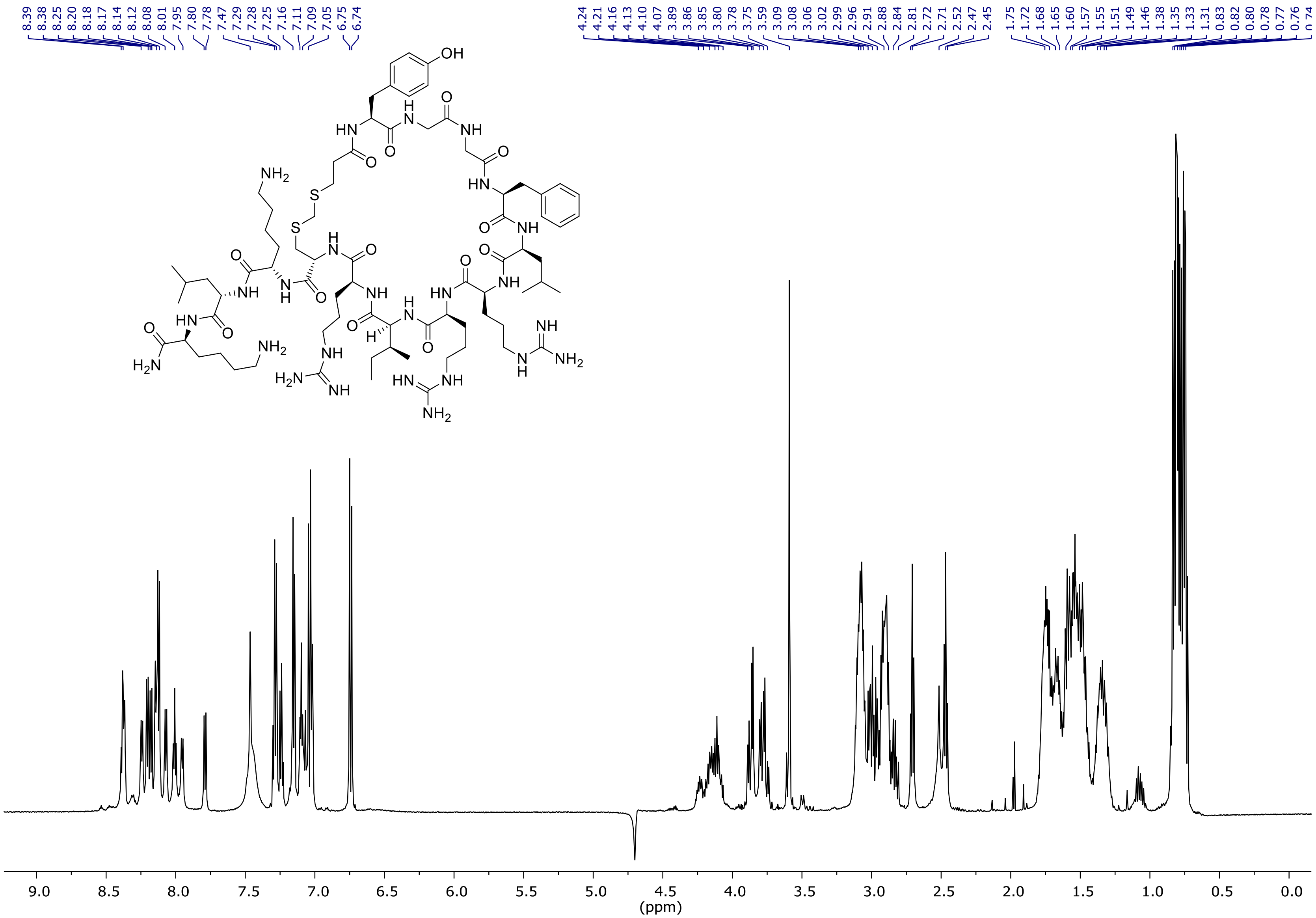


**Figure S23.** $^1H$ **NMR spectrum of CSD-CH$_{2(1,10)}$-NH$_2$ at 600 MHz in H$_2$O/D$_2$O (9:1, v/v)**
